# Water-inrush mechanism from the head-on working face roof in a Jurassic coal seam in the Ordos Basin

**DOI:** 10.1371/journal.pone.0298399

**Published:** 2024-03-12

**Authors:** Longqing Shi, Xingyue Qu, Mei Qiu, Jin Han, Weiqiang Zhang

**Affiliations:** 1 College of Earth Science and Engineering, Shandong University of Science and Technology, Qingdao, China; 2 College of Computer Science and Engineering, Shandong University of Science and Technology, Qingdao, China; 3 Shandong Shengyuan Geological Exploration Co., Ltd, Tai’an, China; Xi’an University of Science and Technology, CHINA

## Abstract

When Chinese coal mines are mining Carboniferous Permian coal seams, the mechanism of water inrush from the roof of the working face usually conforms to the "Upper Three Zones" or "Upper Four Zones" theory. The water inrush passageway is water-conducting fracture zone, and the water inrush position is located in the goaf. However, when mining Jurassic coal seams in Chinese coal mines, the location of water inrush often appears at the head-on working face, above the coal mining machine. Due to the support of the fully mechanized mining support, the roof rock layer cannot collapse and therefore cannot form water-conducting fracture zone. Therefore, the water inrush mechanism cannot be explained by the above two theories. This paper is guided by the Practical Mine Pressure Control Theory, and based on the explanation of the motion forms of bending (pulling) failure movement and shearing (cutting) failure movement, and combined with on-site examples, it is revealed that the passageways leading to from the head-on working face roof in a Jurassic coal seam in the Ordos Basin are splitting zones type, fracture line type and structural fracture type, respectively. Taking the changes in water inflow during the mining process of the 3301 and 3302 working faces in Zhujiamao Coal Mine as examples, this paper reveals the mechanism of water inrush from the head-on working face roof caused by splitting zones type, and proves the existence of this passageway through on-site 3D high-density electrical detection and tracing experiments. Taking two catastrophic water inrush accidents that occurred head-on in the 1309 working face of Guojiahe Coal Industry Co., Ltd. as examples, the water inrush mechanism of the fracture line type and the water inrush mechanism of the structural fracture type were respectively revealed. Based on mechanism of water inrush from head-on roof of working face and the analysis of the on-site water inrush process, a method for distinguishing the type of water inrush passageway from the front roof of the working face is proposed. The results indicate that the Jurassic coal seam mining in the Ordos Basin is prone to shearing (cutting) failure movement, resulting in the frequent formation of the three types of water inrush passageways mentioned above.

## Introduction

Since the establishment of the People’s Republic, China has heavily relied on coal as its primary energy source, constituting around 50%-60% of its energy structure. This reliance on coal is projected to continue for the upcoming decades [1]. In the earlier part of the last century, China’s coal mining activities predominantly centered on the extraction of Carboniferous and Permian coal from the Middle East. However, with the advent of the 21st century, the focus of coal resource development has progressively shifted towards the Jurassic coal reserves located in the central and western regions of China. Among these, the Ordos Basin stands out as a significant coal-bearing region in the country, hosting 31.9% of the assessed Jurassic coal resources [2]. Notably, the Shendong, Northern Shaanxi, Huanglong, and Ningdong regions, all of which exploit Jurassic coal resources within the Ordos Basin, have been approved as large coal bases for mining, supplying considerable coal to northeastern China [3–7]. Moreover, they serve as pivotal coal power suppliers for the northern corridor of the West-East power transmission network in China. Given the Belt and Road initiative’s implementation and the strategic shift towards western coal production, mining of the Jurassic coal seams in this area has emerged as a vital pillar supporting the sustainable growth of China’s coal industry [8–12].

Throughout the course of Jurassic coal-seam exploitation within the Ordos Basin, numerous incidents of water inrush and sand-mud bursts have transpired, causing substantial economic losses and unfortunate human casualties. The overlying strata above the Jurassic coal seams in this region are characterized by their weak cementation, harboring extensive bedrock pore fissure water and Quaternary loose layer water. Scholars have collectively concluded that the underlying mechanism behind catastrophic roof water inrush during Jurassic coal-seam mining in the Ordos Basin revolves around water-conducting fracture zones connecting with rock separation zone water [13–18]. This viewpoint holds practical significance in guiding preventive measures against water-related disasters during Jurassic coal-seam mining in the Ordos Basin.

Nevertheless, through a comparative analysis of roof water inrush accidents between Jurassic coal-seam mining in the Ordos Basin and Carboniferous and Permian coal-seam mining in Northern China, a pronounced divergence emerges: the positions of water inrush differ significantly. While the former involves water inrush from the head-on working face roof, that is, above the coal mining machine on the coal mining face, the latter manifests water inrush occurrences in the goaf situated behind the working face. This distinct variation prompts the investigation to delve into the mechanism underpinning head-on roof water inrush during Jurassic coal-seam mining in the Ordos Basin, employing the Practical Mine Pressure Control Theory as a guiding framework and drawing insights from engineering case studies.

### Basic forms of movement and failure of overlying strata in the stope

In accordance with the principles outlined in the Practical Mine Pressure Control Theory, proposed by professor Song Zhenqi, an esteemed academician of the Chinese Academy of Sciences member, the post-coal mining scenario typically results in two fundamental forms of failure movement within the overlying strata: bending (pulling) and shearing (cutting) [19].

### Bending (pulling) failure movement

**Fig 1:**
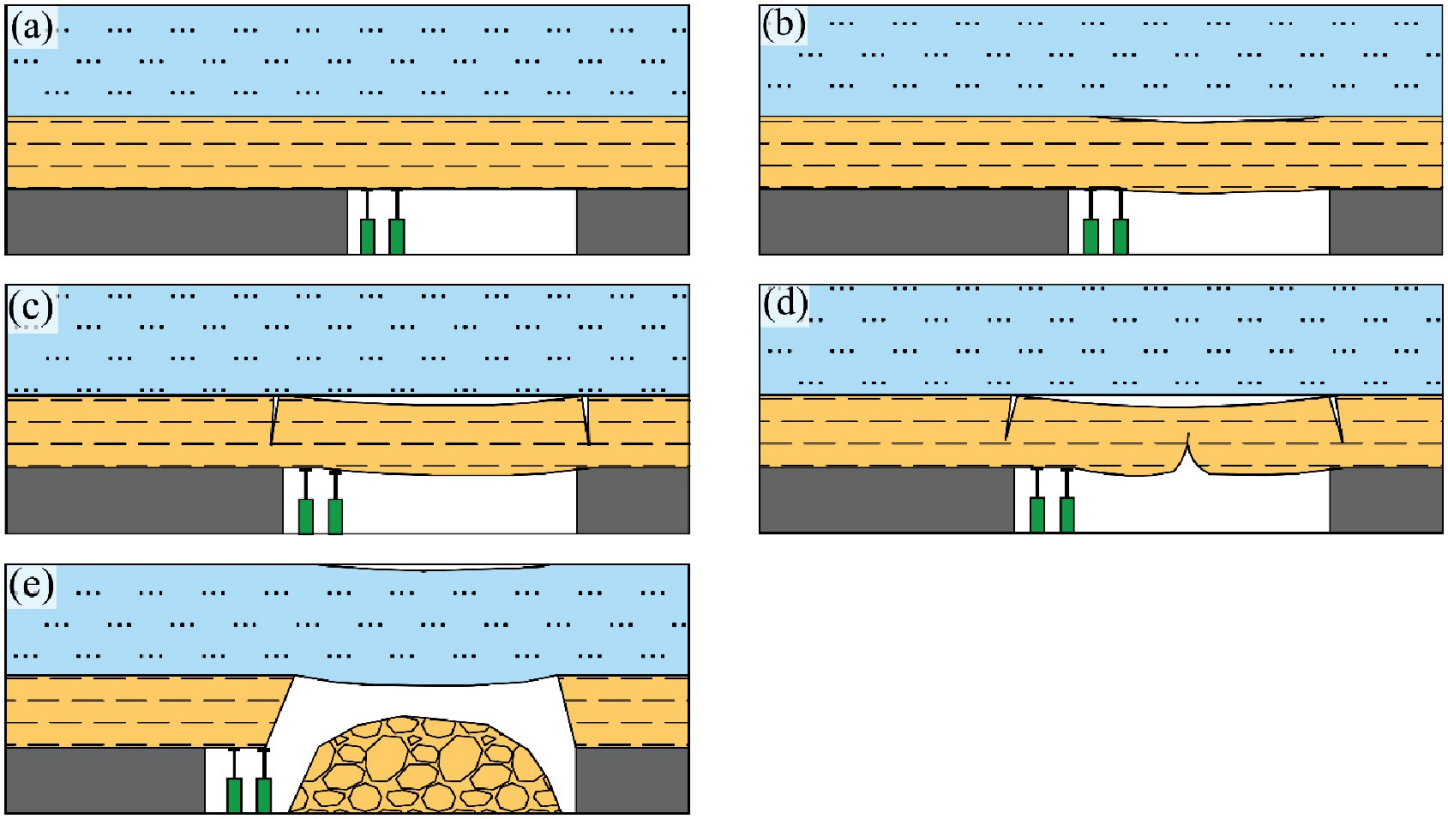
Development of overlying strata bending failure to fall.

As the stope progresses, the overlying strata become progressively exposed (Fig 1a), and they undergo bending due to the influence of gravity (Fig 1b). Subsequently, they are subjected to further exposure over a specific span. As the process of bending and settling advances to a certain threshold, the overlying strata undergo cracking at the extremities that extend towards the coal wall (Fig 1c), and cracks emerge in the middle, resulting in the formation of a pseudo-plastic rock beam (Fig 1d). When the settlement value surpasses the permissible limit of settlement for the pseudo-plastic rock beam, the previously exposed rock stratum experiences a collapse (Fig 1e).

The mechanical condition for developing strata movement from bending settlement to failure is that the maximum bending tensile stress in the strata reaches its tensile strength, described as follows [20]:

$$\sigma_{tmax}=\left[\sigma_{t}\right]$$ (1)

where *σ*_*tmax*_ is the actual maximum tensile stress in the exposed rock stratum, Pa; *σ*_*t*_ is the allowable tensile stress of the suspended rock stratum, Pa.

**Fig 2:**
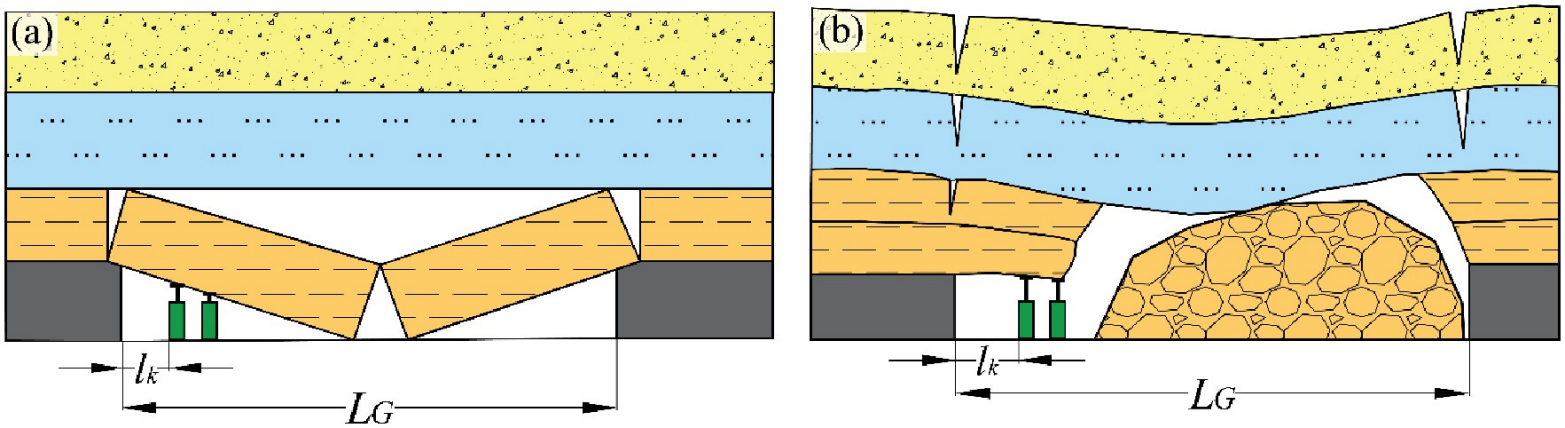
Pseudoplastic rock beam formed by bending failure of the rock stratum.

Once the central portion of the suspended rock is exposed, the decision regarding its failure hinges upon the vertical clearance permitted for movement in its lower section. The transition of rock stratum movement from a state of bending settlement to one of failure will solely occur if the vertical clearance in its lower segment surpasses the permissible settlement limit for the mobile rock stratum. If this criterion is not met, the pseudo-plastic rock beam state, as depicted in Fig 2, will be retained. Consequently, the circumstance under which the bending failure of the *n*th rock stratum situated above the coal seam proceeds to eventual failure is articulated by formula (2) [21, 22].

$$S_{n}>S_{0},S_{n}=h-\sum_{i=1}^{n-1}\;m_{i}\left(K_{A}-1\right)$$ (2)

where *S*_*n*_ is the allowable movement space height of the lower part of the suspended rock stratum, m; *S*_0_ is the allowable settlement value of the suspended rock layer developing into the pseudoplastic rock beam, m; $\sum_{i=1}^{n-1}\;m_{i}$ is the total thickness of the caving strata, m; and *K*_*A*_ is the coefficient of dilatancy of the falling rock.

Conversely, the conditions for maintaining the pseudoplastic rock-beam state after the bending failure of the suspended rock layer are as follows [21, 22]:

$$S_{n}>S_{0}$$ (3)

Under the condition in which the rock stratum develops from bending to failure, the mine pressure in the stope is relatively low because the movement gradually develops. As shown in Fig 2a, to ensure safe rock movement, supports must be able to hold all the weight of the falling rock (direct roof) above the control area. Additionally, the pseudoplastic rock-beam movement must be controlled at the required position. When the pseudoplastic rock beam is allowed to settle to the final position (i.e., without control), the support resistance can be zero, but the maximum rock weight does not have to exceed 1/4 of the rock-beam span, that is, the support resistance *P* can be expressed as follows [23]:

$$A\leq P\leq A+\frac{m_{e}\gamma_{E}L_{0}}{4l_{K}}$$ (4)

where *A* is the support resistance necessary to support the roof, Pa; *m*_*e*_ is the thickness of the pseudoplastic rock beam, m; *γ*_*E*_ is the unit weight of the pseudoplastic rock beam, kN/m^3^; *L*_0_ is the initial fracture step or span of the pseudoplastic rock beam, m; and *l*_*K*_ is the control top distance, m.

According to the bending (pulling) failure movement of the overburden, the failure zone above the goaf is the collapsing suspended rock layer; the fracture zone is the pseudoplastic rock beam.

### Shearing (cutting) failure movement

**Fig 3:**
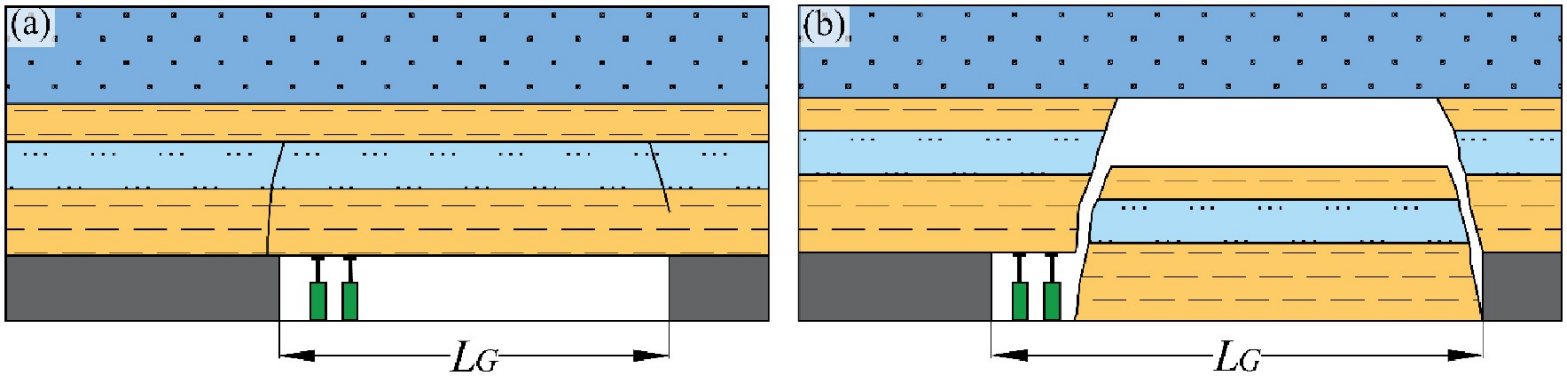
Overlying strata shearing failure movement.

As the stope progresses, a minor degree of bending transpires once the rock stratum is unveiled, resulting in the development of cracks at the extremity of the exposed rock stratum (Fig 3a). In cases where the central region of the rock stratum experiences negligible or limited cracking, the entire stratum is subject to shearing and subsequently collapses (Fig 3b).

The prerequisites for the shearing of suspended rock strata are as follows: When the stope approaches the position where cracks develop at the end of the rock beam, the shear stress acting on the remaining section surpasses its tolerable limit. Despite the middle portion not undergoing cracking, shearing of the rock stratum ensues as long as there is available movement space in the lower segment of the rock stratum.

**Fig 4:**
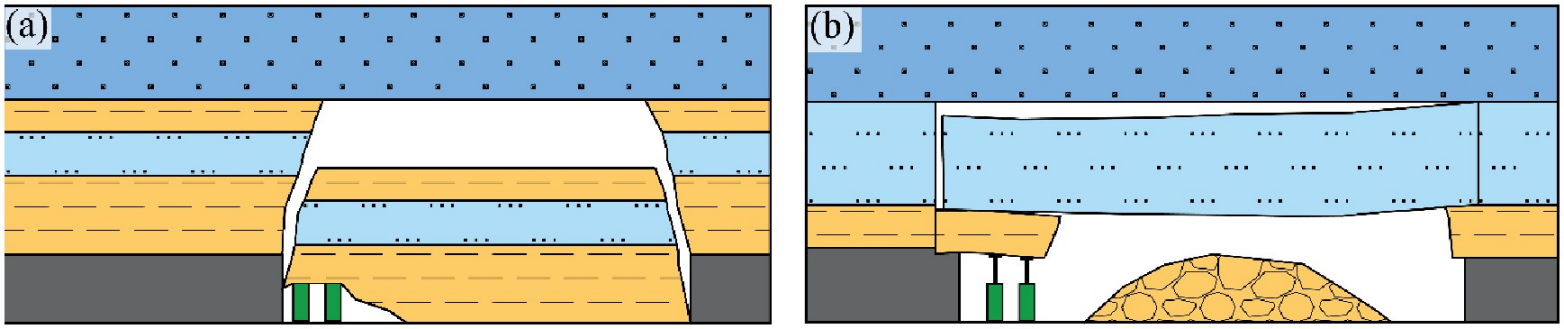
Shearing movement threats to the working face.

This form of failure induces significant dynamic pressure impacts on the stope due to the rapid shearing movement of the rock stratum. In such instances, if the support resistance proves inadequate, a substantial roof collapse incident can readily occur as the roof is sheared along the coal seam (Fig 4a). Even when the stope roof remains intact, a gradual subsidence takes place (Fig 4b), rendering the extraction of support remarkably challenging.

Therefore, to control the overall shearing of the stope roof, the stope must have sufficient support, and its working resistance should prevent the roof from shearing along the coal seam and push the shearing line beyond the control roof distance. The support shrinkage can be considered according to the progressive sinking at the coal seam and the pillar will not be crushed. For the case shown in Fig 4b, the estimated support strength *P*_*T*_ and the rated reduction of the column *ε* are as follows [24, 25]:

$$p_{T}\geq A+\frac{m_{K}\gamma_{K}L_{G}}{2l_{K}}$$ (5)

$$\varepsilon \geq \text{h}-m_{K}\left(K_{A}-1\right)$$ (6)

where *m*_*K*_ is the thickness of the rock stratum that may be cut off as a whole, m; *γ*_*K*_ is the average unit weight of the rock stratum that may be cut off as a whole, kN/m^3^; *L*_*G*_ is the limit span or cutting step of rock stratum that may be cut off as a whole, m. The other variables are as defined previously.

**Fig 5:**
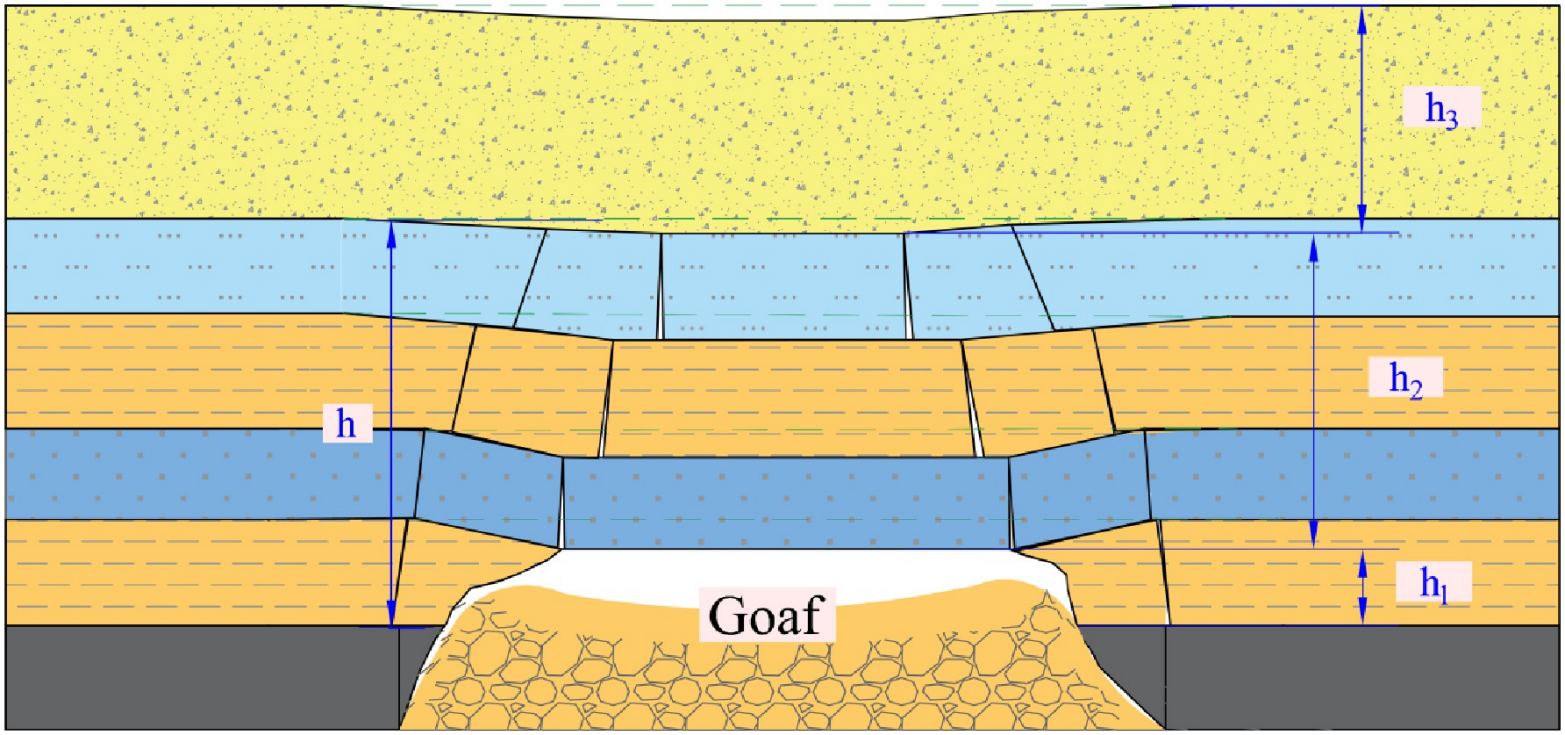
Broken strata upper three-zone model over goaf. *h*_1_: failure zone; *h*_2_: fracture zone; *h*_3_: bending zone; *h*: water-conducting fracture zone.

**Fig 6:**
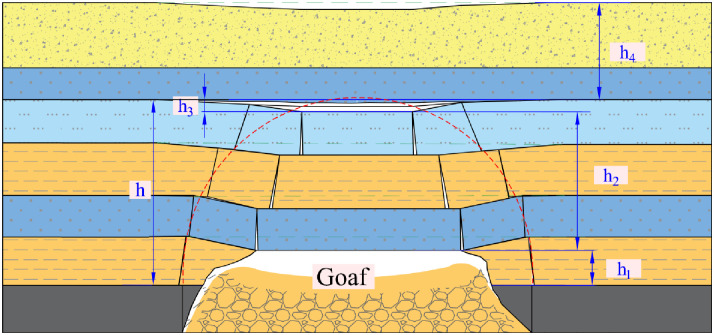
Broken strata upper four-zone model over goaf. *h*_1_: failure zone; *h*_2_: fracture zone; *h*_3_: rock-separation zone; *h*_4_: bending zone; *h*: water-conducting fracture zone.

Based on the aforementioned points, the following two insights can be deduced:

Distinct Formation of Zones: The creation of the “Upper Three Zones” [26, 27] (as depicted in Fig 5) or the “Upper Four Zones” (as illustrated in Fig 6) [28, 29] is exclusively associated with the movement failure pattern of bending (pulling). Conversely, the broader section of collapsed rock strata stemming from the shearing (cutting) failure lacks the capacity to give rise to these particular zones.

In the early 1980s, represented by Academician Liu Tianquan, former scientists and technicians from the Beijing Mining Research Institute of the General Institute of Coal Science and Technology proposed the "Upper Three Zones" theoretical model when studying the laws of coal mine surface movement and overlying rock failure. Under the conditions of longwall caving mining, the most common form of rock deformation around coal seams is the "Three Zones" type. This model divides the overlying strata in the goaf into failure zone, fracture zones and bending zone. (referred to as the "Upper Three Zones" theory).

In order to apply the numerical analysis method of rock mechanics to calculate the movement of rock layers and surface, Professor Gao Yanfa proposed a "Four Zones" division model for the roof of goaf in coal mining. Professor Shi Longqing combined the "Upper Three Zones" theory and the "Four Zones" division model to propose a "Upper Four Zones" theoretical model for roof water hazard prevention and control research. This model divides the overlying strata in the goaf into failure zone, fracture zones, rock-separation zones, and bending zone (referred to as the "Upper Four Zones" theory).

Zone Models and Roof Water Inrush: Both of the abovementioned zone models are specifically designed to address the roof water inrush scenario concerning the goaf located behind the working face.

### Mechanism of water inrush from the working face head-on roof

#### Water inrush from passageways caused by overlying strata shearing (cutting) failure

Due to the shearing (cutting) failure, fracture lines tend to emerge extending upwards along the extremities of the overlying strata. These fracture lines, once they reach aquifers or water-bearing formations, serve as conduits for water inrush originating from the head-on roof of the working face. According to the practical principles of mine pressure control, although shearing (cutting) failures in the overlying strata are more common in shallow coal-seam mining, they can also occur during deep coal-seam mining when the overlying strata exhibit weak cementation.

#### Mechanism of water inrush from the head-on working face roof caused by splitting zones

Roof water inrush due to splitting zones arises when the overlying strata possess weak cementation, and fracture lines produced by shearing (cutting) failure extend up to the surface, particularly in shallow coal-seam mining. These fracture lines, commonly referred to as splitting zones, serve as pathways that not only link the overlying aquifer with the head-on roof of the working face or the goaf but also allow surface water to infiltrate the underground stope. This connection with surface water results in the formation of a splitting-zone roof water inrush.

Illustrating the mechanism using a specific case, the disastrous water inrush incident from the head-on roofs of the 3301 and 3302 mining faces within the Zhujiamao coal mine (Shaanxi ChinaTye Energy Co., Ltd) highlights the mechanism of water inrush from the head-on working face roof caused by splittingzones.

**Fig 7:**
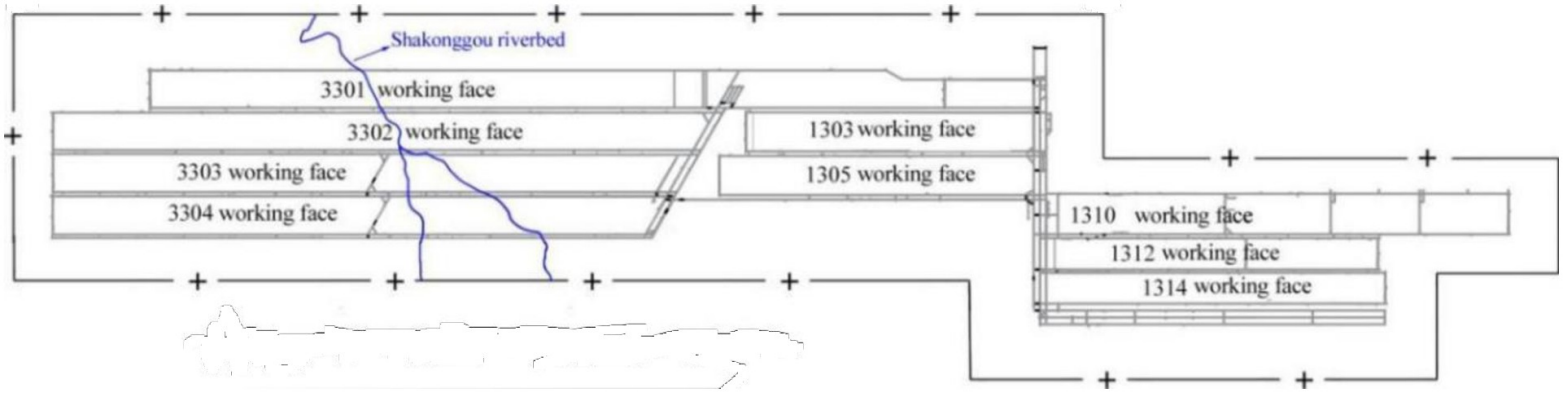
Geographic location of the Zhujiamao coal mine.

**Fig 8:**
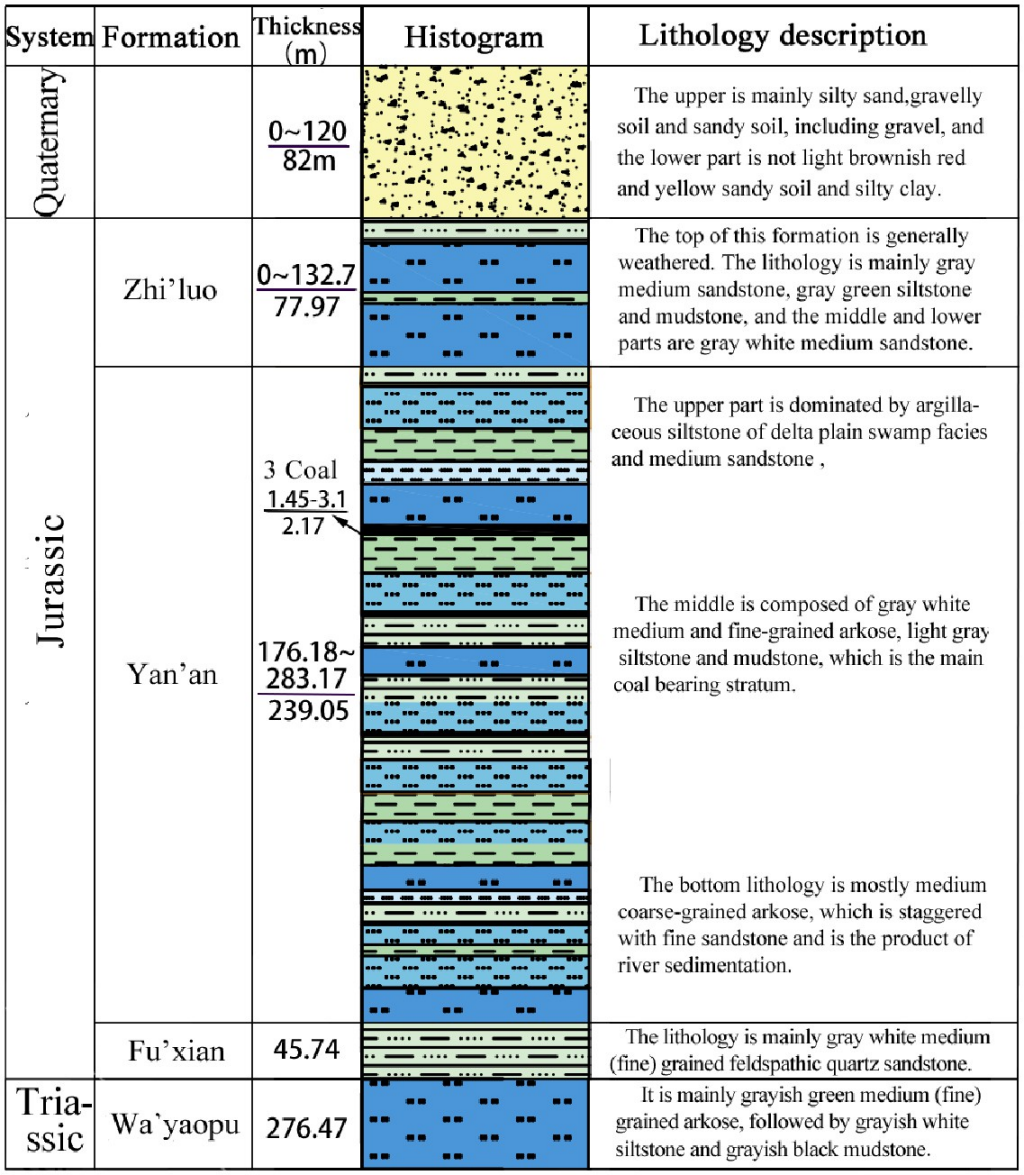
Stratum histogram of the Zhujiamao coal mine.

Geological context in the Zhujiamao coal mine: The Zhujiamao coal mine is situated in the northeastern part of the Yuheng mining area (South Zone, Jurassic Coalfield) in northern Shaanxi, approximately 20 km away from Hengshan County. Administratively, it falls under the jurisdiction of Boluo town and Dianshi town (Fig 7). The mining area spans roughly 4.7–5.3 km from north to south and 5.2–10.0 km from east to west, covering an expanse of 50.1932 km^2^. The region predominantly features Quaternary loose sedimentary layers, with the Upper Triassic Wayaobu Formation (T_3_w), the Lower Jurassic Fuxian Formation (J_1_f), the Middle Jurassic Yan’an (J_2_y), and Zhiluo (J_2_z) formations, as well as Quaternary (Q) strata, sequentially exposed (Fig 8). Notably, no significant faults or folds have been identified within the Zhujiamao coal mine to date. The coal deposit is characterized by a simple monoclinic structure gently dipping westward, exhibiting a dip direction of 277° and a dip angle of 0.6°, with no associated magmatic activity. The Yan’an Formation (J_2_y) constitutes the primary coal-bearing strata, ranging in thickness from 188.27 m to 251.59 m, and averaging 232.46 m. Within this stratum, seven to nine coal seams are present, numbered 8, 6, 5, 4–1, 4, 3–1, and 3 from bottom to top. Among these, only seam No. 3 is suitable for mining.

**Fig 9:**
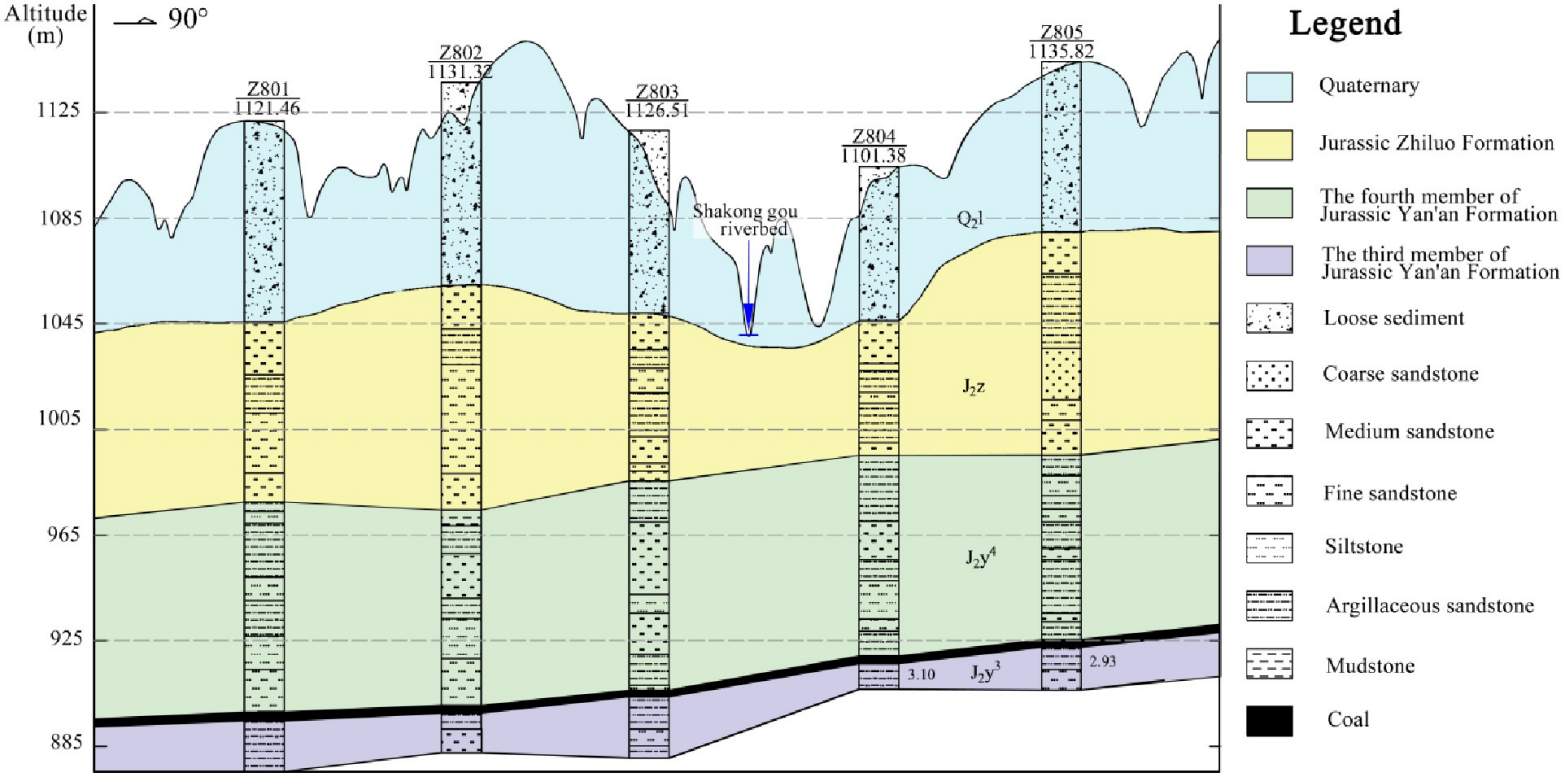
Along-strike profile of the 3302 working face in the Zhujiamao coal mine.

Water-inrush mechanism for the 3301 and 3302 working faces in Zhujiamao coal mine: The 3301 working face was the initial extraction area within the No. 3 coal seam of the Zhujiamao coal mine. Its working face span, denoting the width of the stope, measures 250 m, and it spans a length of 3620 m. Subsequently, the 3302 working face followed, encompassing a stope width of 210 m and a length of 4050 m. The mining technique employed involves full-seam mining, and the roof is managed using the fallen method. The strata exposed as a result of drilling activities in the 3302 working face are visually represented in Fig 9.

**Fig 10:**
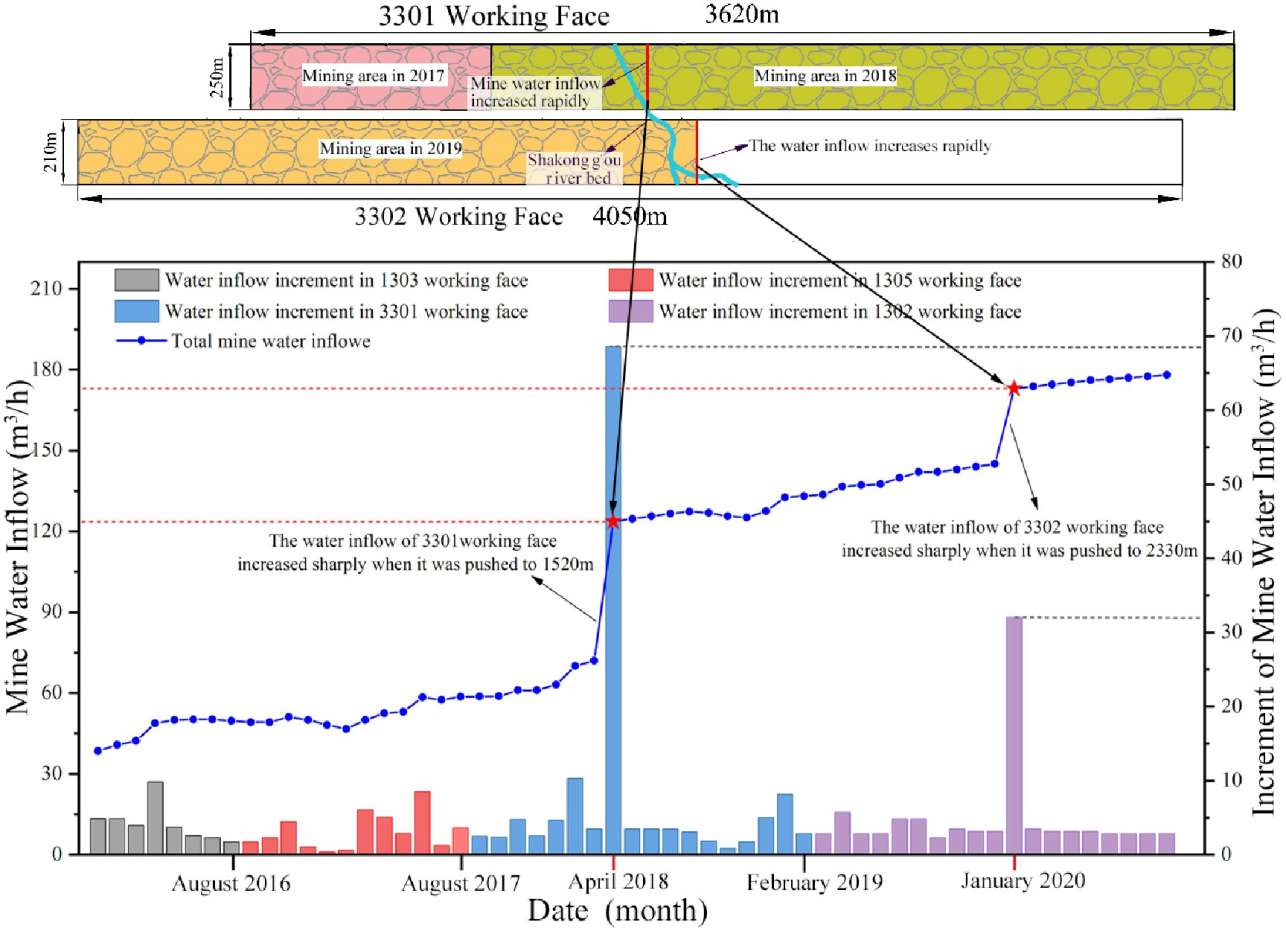
It illustrates the positions of head-on water inrush occurrences for the 3301 and 3302 working faces within the Zhujiamao coal mine, along with the curve depicting the trends in mine water inflow.

The mining operations of the 3301 working face commenced on August 24, 2017. Before December 2017, only a minimal amount of water was observed dripping from the head-on working face roof, resulting in a mine water inflow of less than 60 m³/h. However, on April 1, 2018, when the working face had advanced to a distance of around 1520 m, the water inrush discharge from the head-on working face roof escalated to about 60 m³/h, correspondingly raising the mine water inflow to 125 m³/h. Subsequent to this occurrence, the mine water inflow gradually increased as the working face continued to advance. Eventually, the 3301 working face was fully mined out on February 26, 2019, and by that point, the mine water inflow had reached approximately 133 m³/h (Fig 10).

On January 1, 2019, the mining operations for the 3302 working face were initiated. Up until July 2019, only a minor quantity of water was observed dripping from the head-on working face roof, leading to a mine water inflow of less than 140 m³/h. Following this period, as the 3302 working face progressed, the mine water inflow gradually increased. It is important to note that during this period, the water inflow from the head-on roof did not exhibit noticeable increments. The stable water dripping from the head-on roof persisted until December 30, 2019, at an advanced distance of approximately 2330 m. At this juncture, a sudden spike was observed, with the water flow increasing to approximately 30 m³/h, subsequently causing the mine water inflow to rise to approximately 173 m³/h (Fig 10).

**Fig 11:**
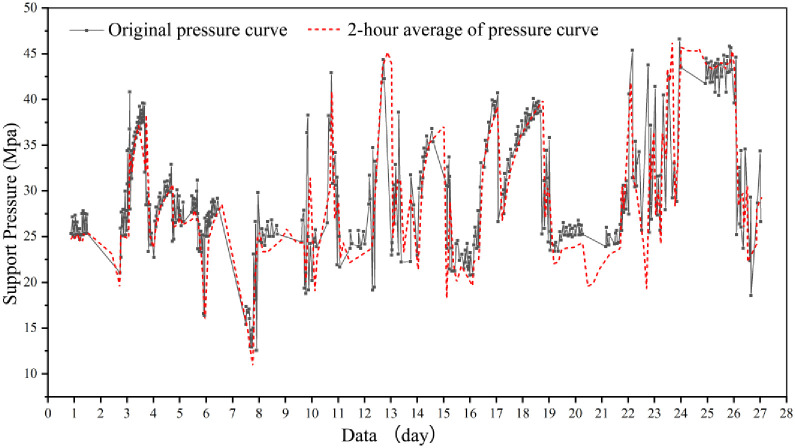
Variation of support pressure over time for the 3301 working face in the Zhujiamao coal mine.

**Fig 12:**
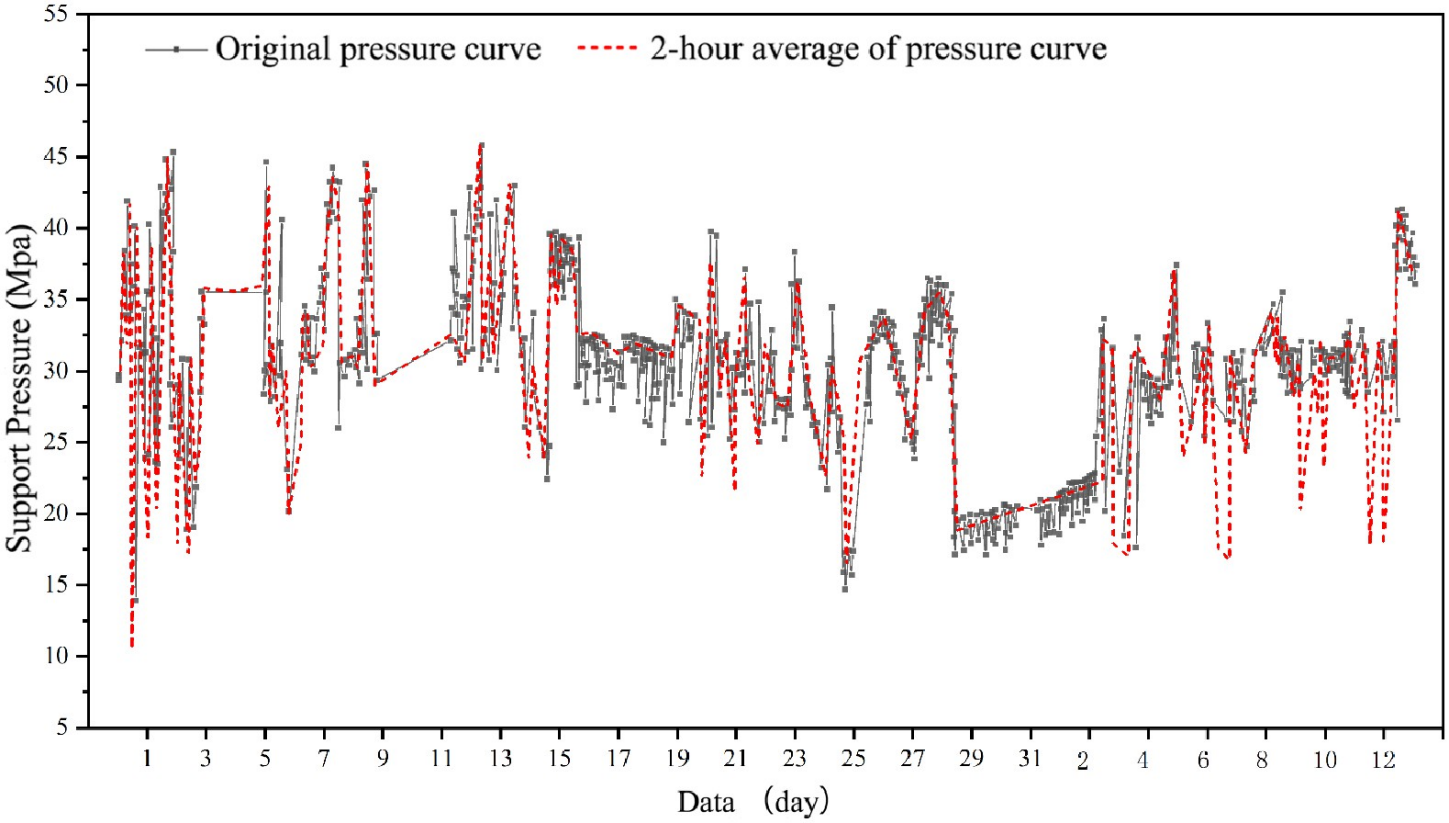
Variation of support pressure over time for the 3302 working face in the Zhujiamao coal mine.

Based on the mine pressure observation data from the 3301 working face (Fig 11) and the 3302 working face (Fig 12), a discernible pulsating pattern is evident in the curves depicting the changes in working support resistance over time. This distinct characteristic, in accordance with the practical principles of mine pressure control, signifies the presence of shearing (cutting) failure within the overlying strata as the working face advances. Consequently, the advancement of the working face within the Zhujiamao coal mine triggers the formation of fracture lines due to the movement of the overlying strata.

The average mining depth of both the 3301 and 3302 working faces is approximately 200 m, while the thickness of the bedrock does not exceed 150 m. This suggests that fracture lines have the potential to extend to the surface, giving rise to the formation of a splitting zone. Furthermore, the surface locations corresponding to the two instances of water inrush from the head-on working face roof are proximate to the Shakonggou riverbed. Consequently, there is speculation that water from the Shakonggou riverbed could infiltrate the underground stope through the splitting zone.

**Fig 13:**
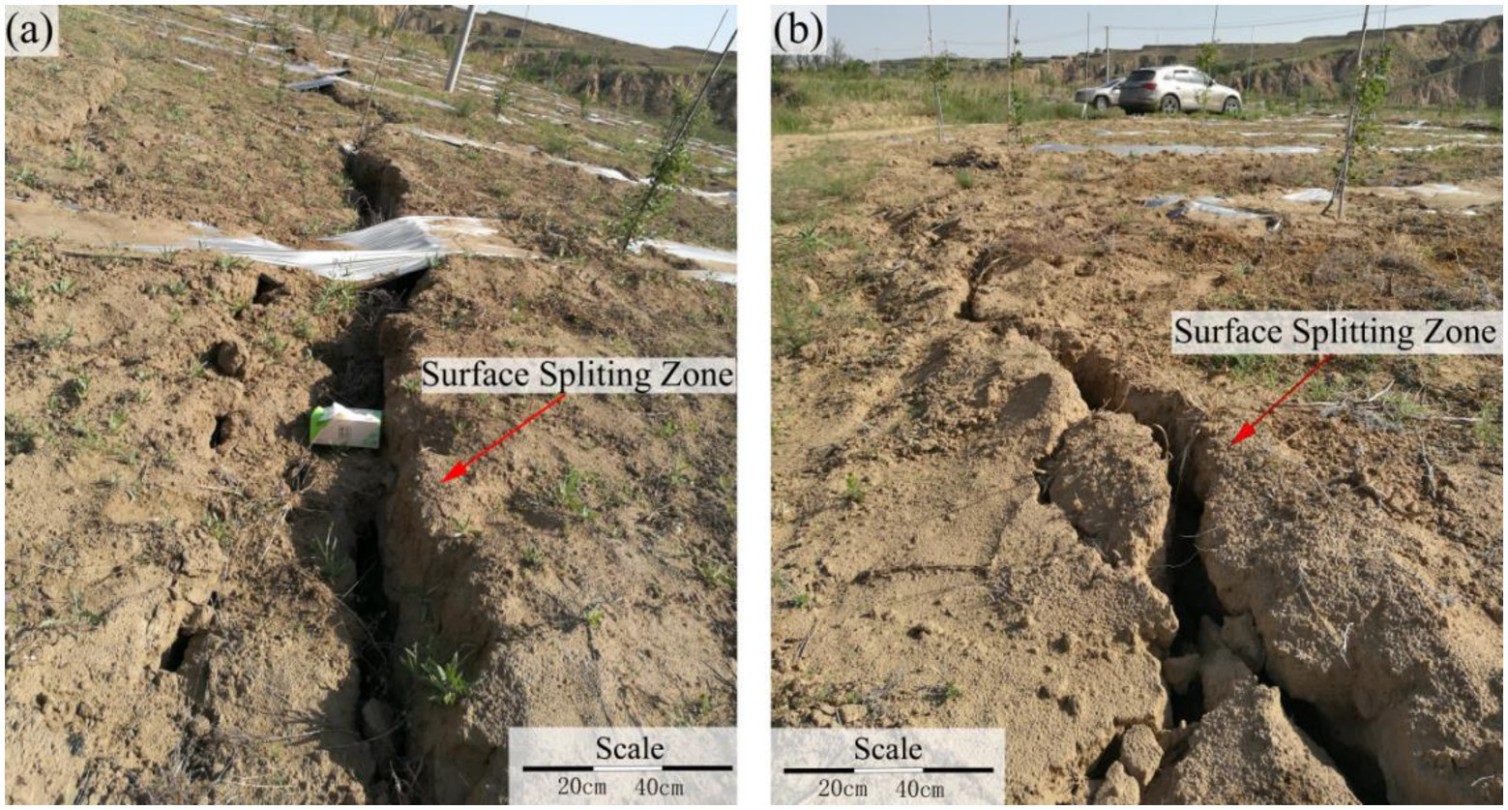
Surface splitting zone in the Zhujiamao coal mine.

**Fig 14:**
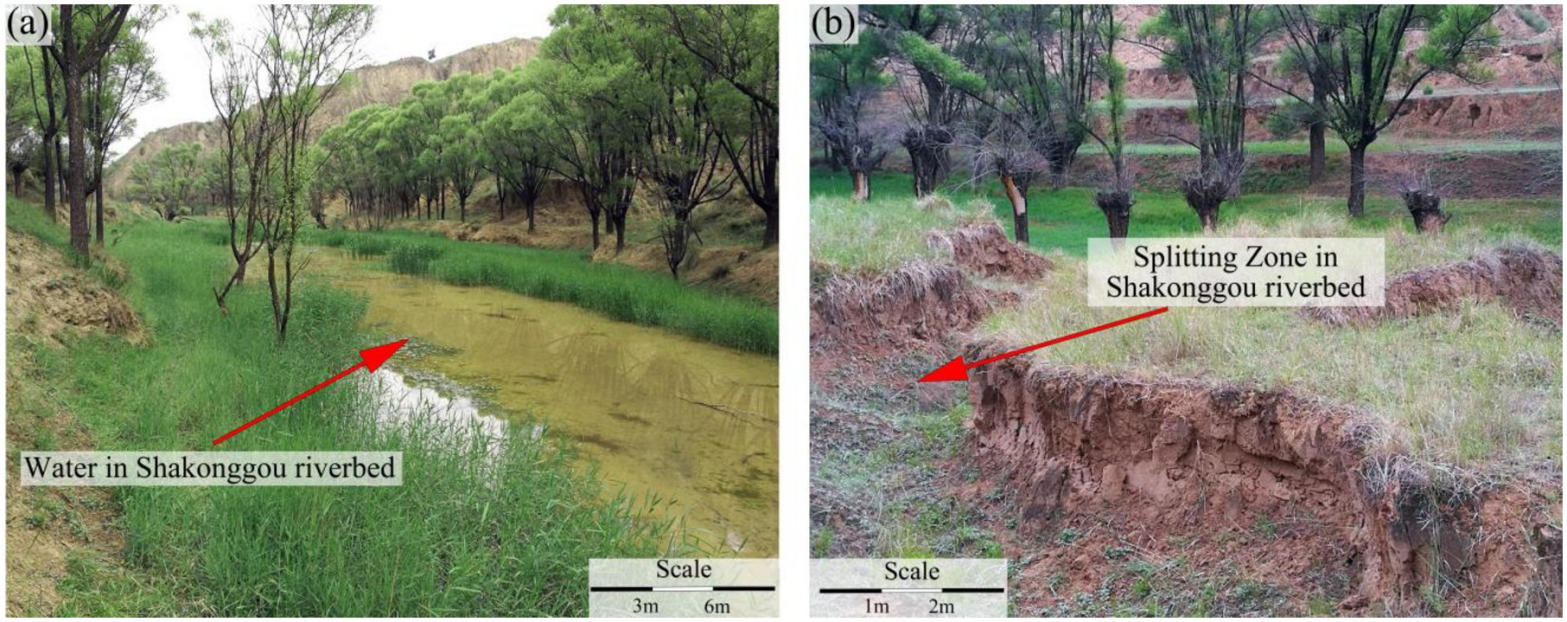
Water and splitting zone in the Shakonggou riverbed.

This speculation prompted a comprehensive field ground survey (Fig 13). The surface splitting zone exhibits a robust development, boasting a width that reaches up to 0.5 m. Importantly, its extension direction aligns with the direction of the working face’s advancement (Fig 13). Subsequent field assessments further revealed that as the 3302 working face progressed continuously to an advanced distance of 2270 m, it intersected a well-formed surface splitting zone that traversed the Shakonggou riverbed-a location characterized by perennial water flow (Fig 14). Consequently, it is plausible to infer that the substantial increase in water inflow (from both the head-on working face roof and the entire mine) can be attributed to the infiltration of water from the Shakonggou riverbed into the underground stope via the splitting zone.

**Fig 15:**
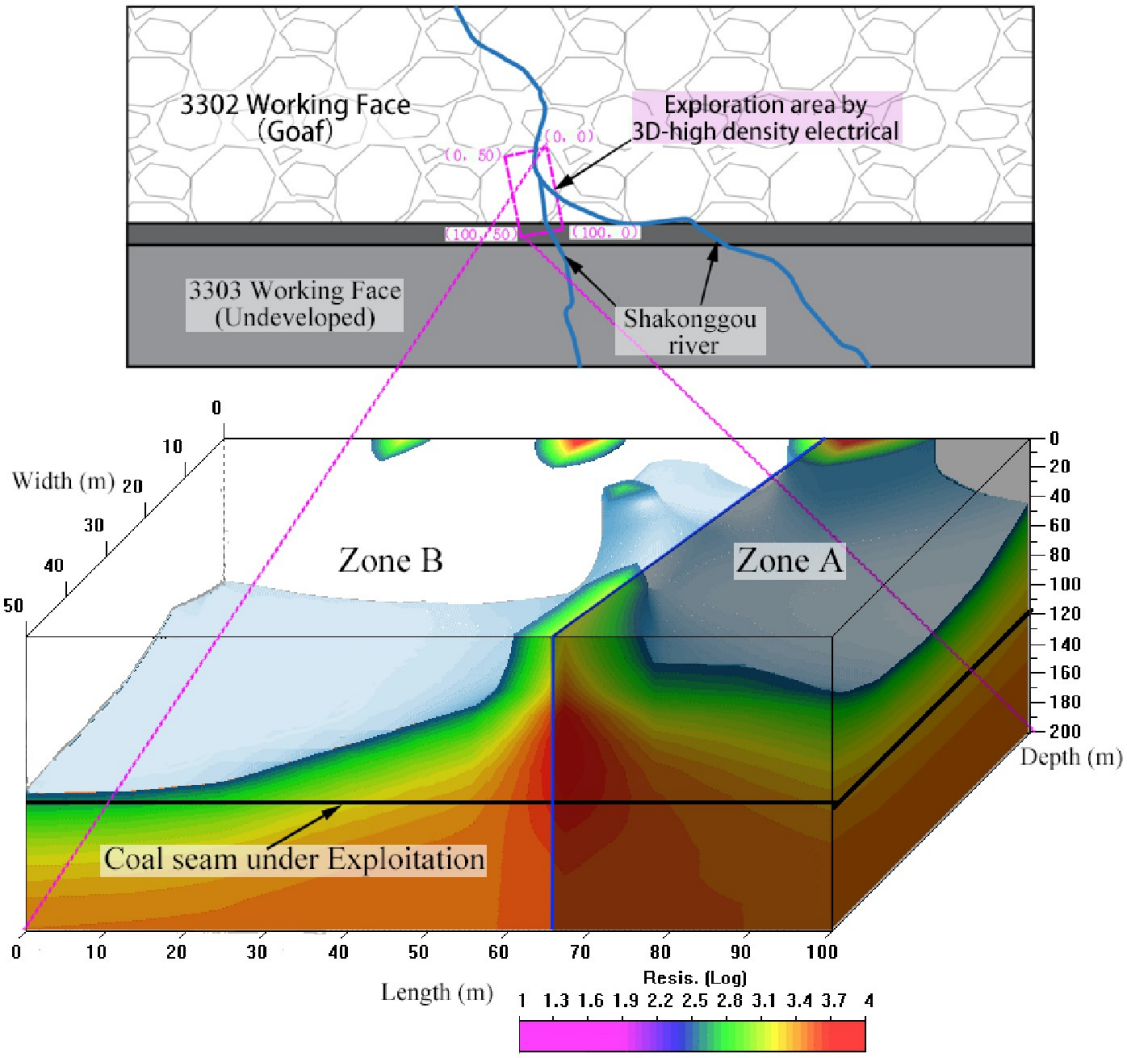
Results of three-dimensional (3D) high-density electrical prospecting in the Shakonggou riverbed.

In order to substantiate this inference, a comprehensive three-dimensional (3D) high-density electrical prospecting was conducted within the Shakonggou riverbed (Fig 15). The outcome of this geophysical prospecting effort unveiled that Zone B corresponds to the area where the splitting zone is extensively developed, whereas Zone A pertains to an area where the coal seams remain untouched. The surface water present in the Shakonggou riverbed holds the capability to penetrate the goaf and the head-on working face roof through the splitting zone found within Zone B. This phenomenon contributes to the continual escalation of mine water inflow.

To enhance the credibility of the geophysical prospecting outcomes, a tracer testing using potassium iodide (KI) was implemented within the region identified by the 3D high-density electrical prospecting. The tracer substance cannot be directly introduced into the water; rather, it is buried in proximity to ensure a gradual and sustained release. The tracer material is buried within a moistened pit.

**Fig 16:**
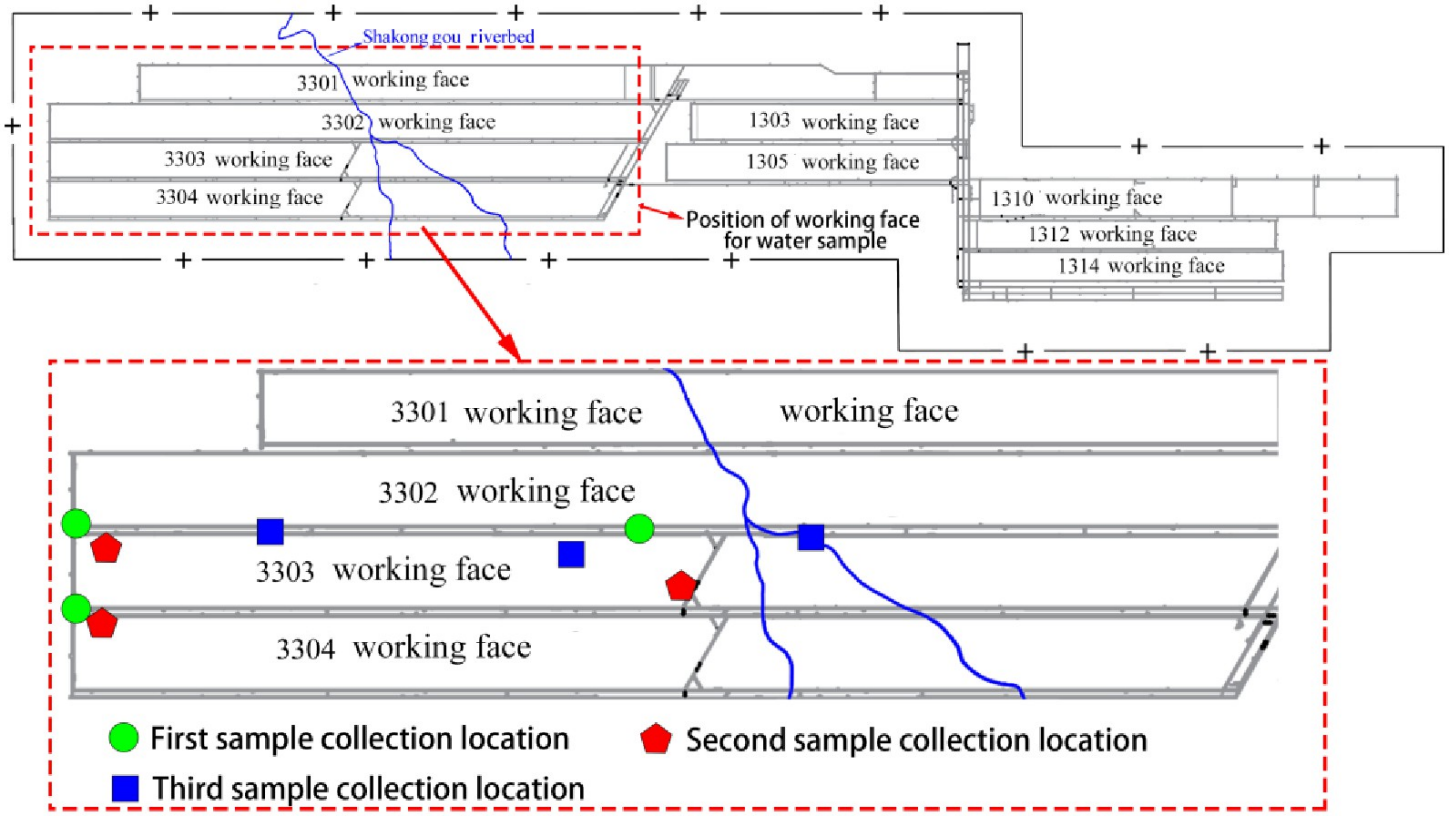
Sketch map for collecting positions of different batches.

The 3D high-density electrical survey and tracer deposition took place on July 5, 2020. Subsequently, water samples were gathered from various inflow points on August 9, September 8, and October 10, 2020 (as depicted in Fig 16), encompassing a broad spectrum of water inflow conditions. Different batches of samples are delineated using distinct colors, serving to differentiate between the various collection instances.

The water samples collected during the initial batch (18) are denoted by the color red, the second set of samples (20) is represented in green, and the third set (16) is depicted in blue. These collected samples were subjected to iodide analysis through ion chromatography (utilizing DIONEX AQUION Ion Chromatography), with the method’s detection limit set at 0.002 mg/L. The testing outcomes are presented in S1 Table. In the first set of water samples, iodide was not detected, likely due to the time required for iodide-containing surface water to permeate into the mine. As time progressed, a significant increase in both iodide concentrations and the proportion of samples containing iodide was observed. This trend in the tracing results supports the notion that the link between surface water and the splitting zone serves as the primary factor contributing to the ongoing elevation of mine water inflow and water inrush from the head-on working face roof. The tracer testing not only validates the precision and dependability of the findings obtained through the 3D high-density electrical survey but also substantiates the inference that surface water from the Shakonggou riverbed is indeed capable of entering the stope through the splitting zone.

From S1 Table, it can be seen that no tracer was detected in the first batch of samples, indicating that surface water cannot penetrate the coal working face through the splitting zones after about 30 days. The second batch of samples detected tracers, indicating that between 30 and 60 days, surface water can penetrate into the coal working face through the splitting zones. The tracer concentration detected in the third batch of samples was significantly higher than that in the second batch, indicating a gradual increase in the permeability of the crack line.

#### Mechanism of water inrush from the head-on working face roof caused by fracture lines

In instances of deep coal-seam mining, if the overlying strata possess weak cementation, the fracture lines induced by shearing (cutting) failure might not extend upwards to the surface. Instead, they could terminate at the base of harder strata. In the context of the Jurassic coal stratum, these harder strata often comprise conglomerates, which are inherently water-bearing rocks. Consequently, fracture lines hold the capacity to not only channel water from the water-bearing rocks they intersect into the head-on working face roof but also to transport water from the conglomerates where the fracture lines culminate into the head-on working face roof.

To illustrate this mechanism, let’s consider the water inrush event from the head-on roof of the 1309 working face at the Guojiahe Coal Industry Co., Ltd. mine in Shaanxi, occurring from August 2 to August 21, 2020. This example serves to elucidate the mechanism of roof water inrush through passageways formed by fracture lines during coal-seam mining.

Geological setting in the Guojiahe coal mine: The Guojiahe coal mine is situated within the Yonglong mining area, which is part of the Jurassic Huanglong Coalfield in Shaanxi Province. Administratively, it falls under the jurisdiction of Tiantang town, Zhangba town, and Zhaoxian town within Linyou County, Baoji City. The mine area spans approximately 14.8 km from east to west and around 8.4 km from north to south, covering a total expanse of 94.6166 km^2^. The mining method employed is fully mechanized caving mining, and the roof is managed using the fallen method.

**Fig 17:**
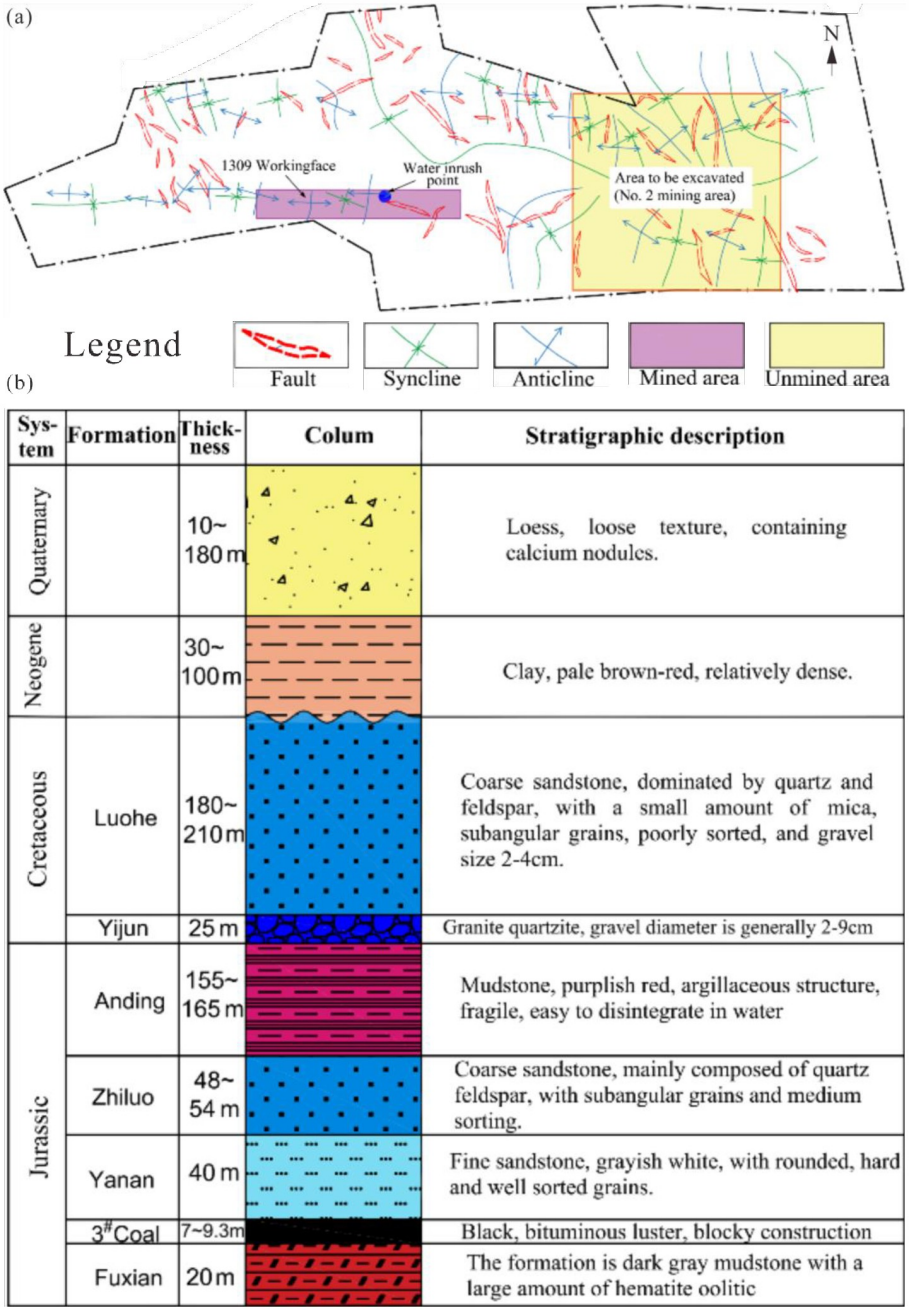
Sketch map for location, structures, and strata in the Guojiahe coal mine.

The geological formations present in the area encompass the Upper Triassic Tongchuan Formation (T_2_t), Lower Jurassic Fuxian Formation (J_1_f), Middle Jurassic Yan’an (J_2_y), Zhiluo (J_2_z), and Anding (J_2_a) formations, Lower Cretaceous Yijun (K_1_y) and Luohe (K_1_l) formations, Upper Tertiary (N) deposits, Pleistocene layers (Q_2-3_), and Holocene series (Q_4_), arranged chronologically from old to new. The geological structure is characterized by a monoclinic orientation towards the northeast, with a northwest dipping angle. The region features mild folds and tensional fracture structures, and magmatic activity is absent. The primary coal-bearing stratum is the Middle Jurassic Yan’an Formation (J_2_y), housing the Number 3 and Number 2 minable coal seams, with coal seam No. 3 being the principal minable coal seam. This seam exhibits an exploitable thickness ranging from 1.10 m to 26.83 m (refer to Fig 17).

The 1309 working face within the Guojiahe Coal Industry Co., Ltd. mine stretches across a length of 2274 m and boasts a width of 225 m. The coal seams in this area exhibit thicknesses ranging from 7 m to 15 m, and the mining operations are carried out at an approximate depth of 540 m. On December 30, 2020, the cumulative mining distance for the 1309 working face amounted to approximately 982 m. At this point, the working face was situated roughly 18 m ahead of the anticline axis, in the direction of working face advancement. It was on this date that the mine encountered a sudden and substantial increase in mine pressure, resulting in the complete collapse of all fully mechanized mining supports. Coinciding with this event, water inrush transpired from the head-on working face roof.

**Fig 18:**
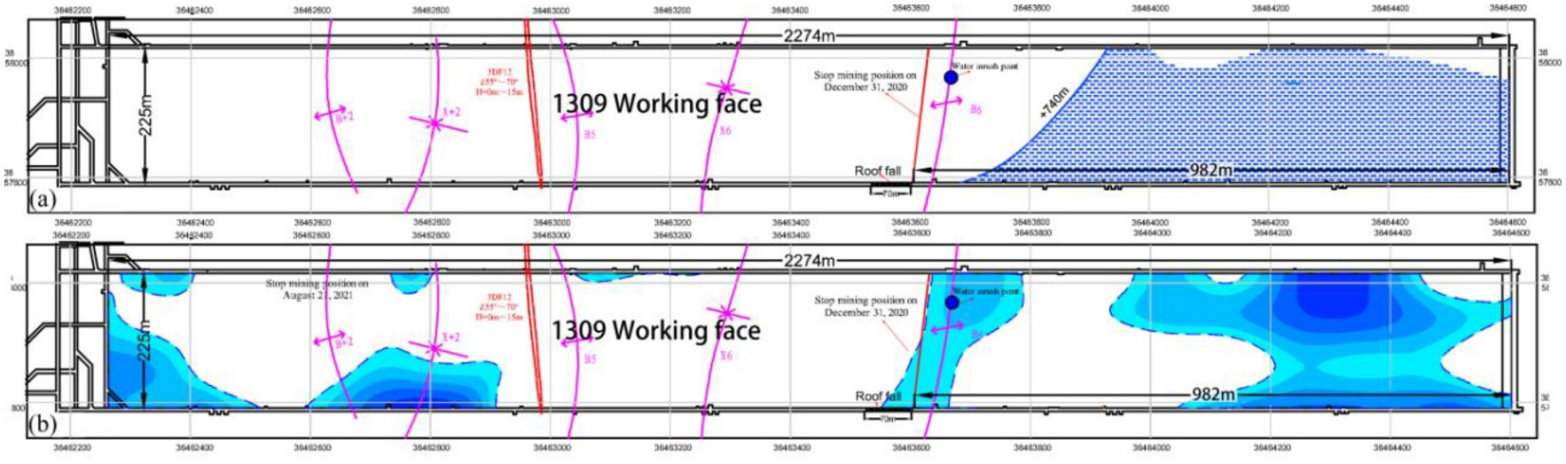
Water-inrush point in the 1309 head-on working face in the Guojiahe coal mine, December 30, 2020.

At the time of this incident, the working face was located downhill, and the mined coal seam measured around 10.3 m in thickness. The ensuing collapse event (reaching heights of up to 70 m) manifested in the lower gangway of the working face (Fig 18). This water inrush episode extended over approximately 5 days, with the peak water inflow rate reaching approximately 500 m^3^/h. The cumulative water inflow amounted to around 43,045 m^3^, and a substantial volume of large rock fragments and gravels surged into the mine. Throughout this process, the water exhibited turbidity and a pale color. The pronounced economic losses incurred due to this incident, which rendered the fully mechanized mining supports irrecoverable, necessitated the reopening of the open-off cut.

Water quality data provide insight into the primary sources behind this roof water inrush, revealing that the primary contributors are the conglomerate aquifer within the Yijun Formation and the sandstone aquifer within the Luohe Formation.

**Fig 19:**
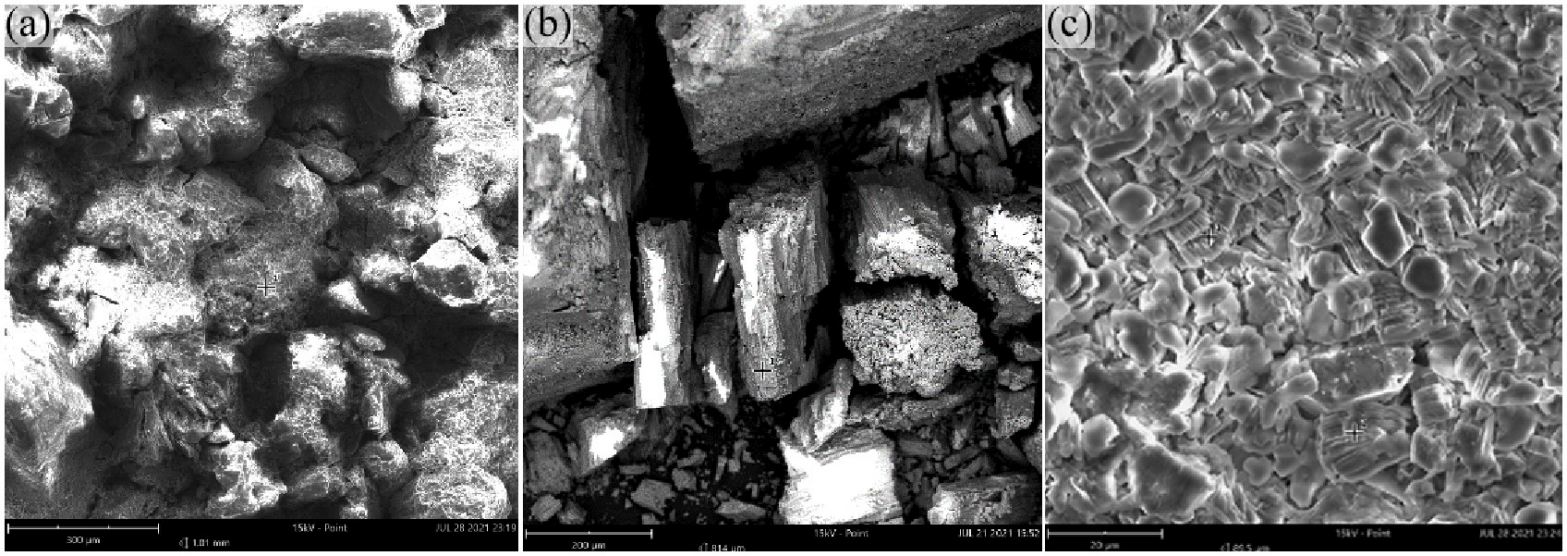
Scanning electron microscopy images for Jurassic sandstones.

Water-inrush mechanism for the 1309 working face: The channels through which the water-inrush event occurred were formed by fracture lines resulting from shearing (cutting) failure in the overlying strata. An examination of Fig 17 reveals that the strata above the No. 3 coal seam, including the Yan’an and Zhiluo formations, consist mainly of sand-mud interbeds. Within the Anding Formation, sandstone and mudstone layers alternate in the middle, while the upper and lower portions are predominantly mudstone. The upper mudstone layer is over 60 m thick, according to drilling data. Scanning electron microscopy (SEM) findings indicate that rock fragments in the Jurassic Yan’an, Zhiluo, and Anding formation sandstones are primarily composed of quartz (Fig 19a) and feldspar (Fig 19b), with a lesser presence of mica. The key cement in the weakly cemented sandstone is montmorillonite (Fig 19c). Triaxial testing underscores the very low shearing strengths of these sandstones (S2 Table).

**Fig 20:**
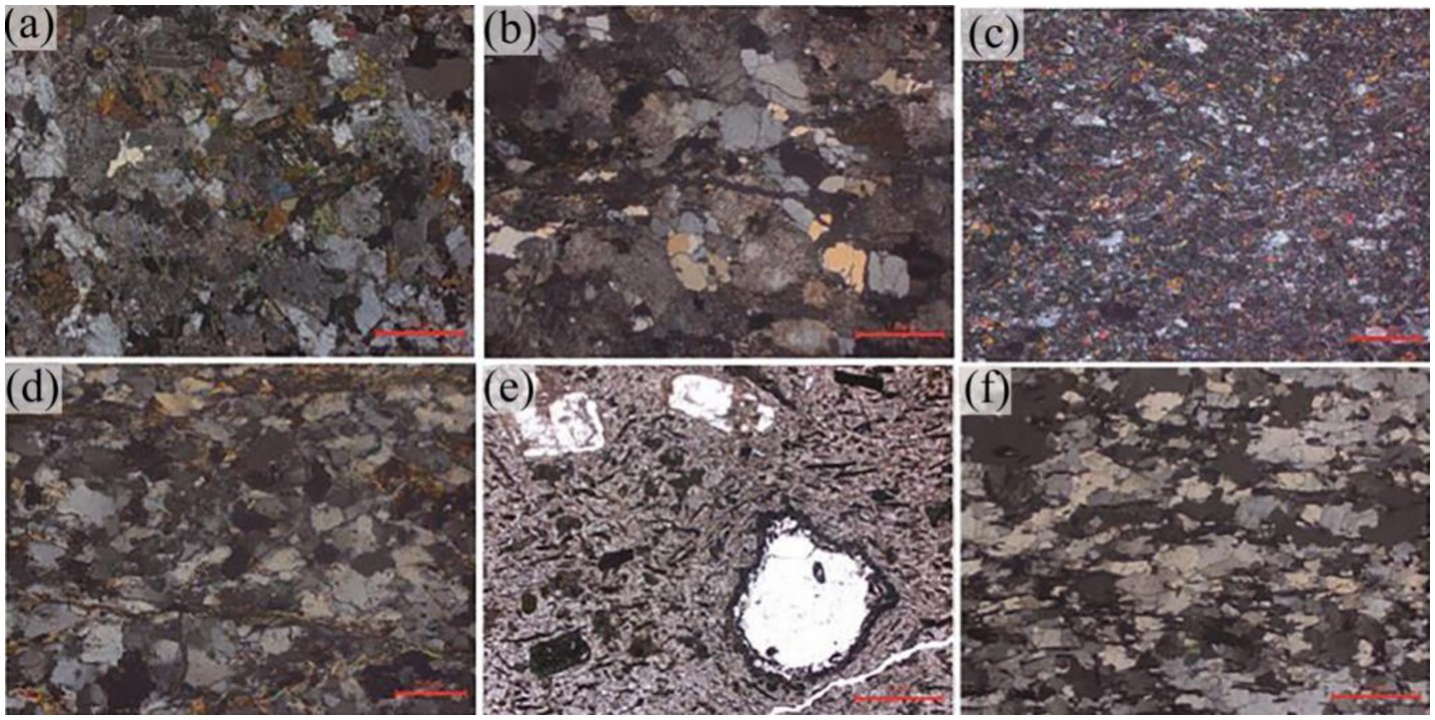
Micrographs of lower Cretaceous Yijun formation conglomerate in the study area (according to Yu [30]). (a) Monzonitic granite; (b) Granodiorite; (c) Epidote chlorite metamorphic andesite; (d) Quartz sandstone; (e) Rhyolite; (f) Gneiss.

**Fig 21:**
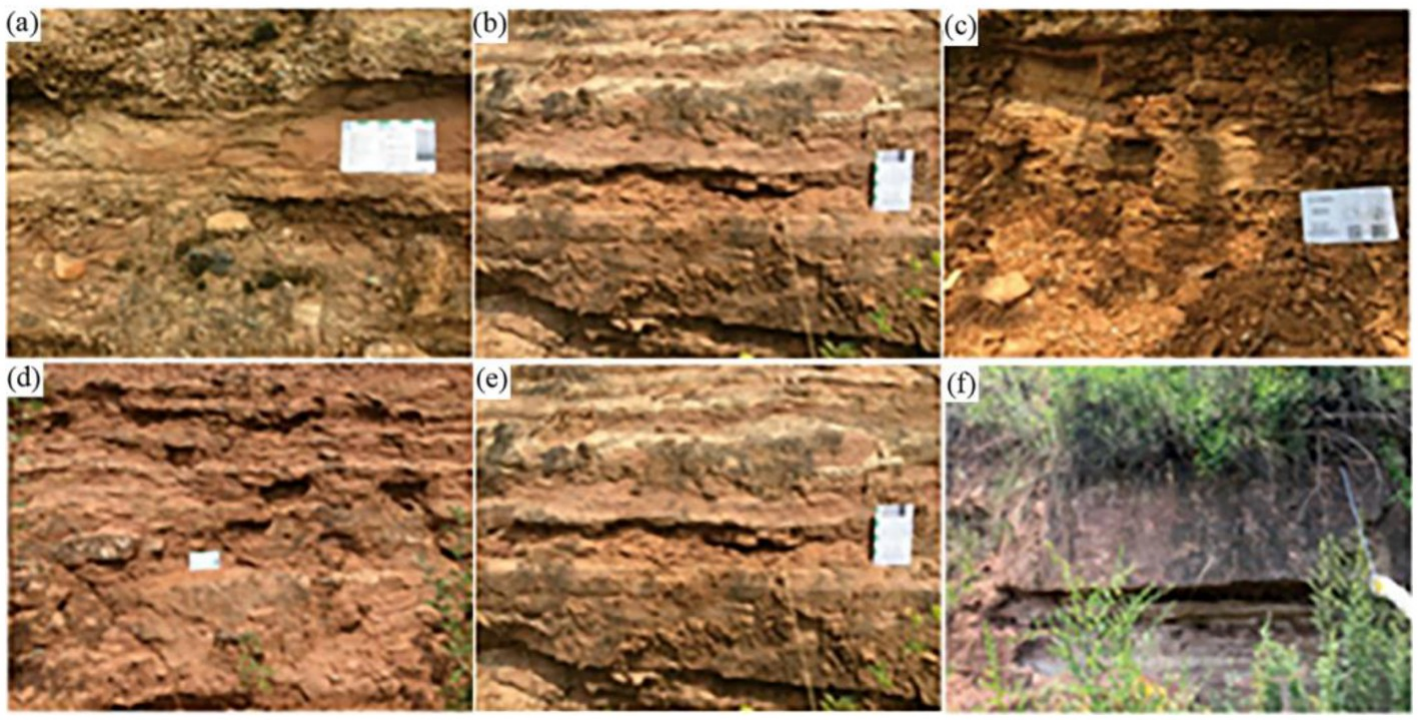
Field characteristics of the Luohe Formation sandstone in the study area (according to Yu [30]). (a) Medium-fine sandstone in the lower Luohe Formation; (b) Sandstone intercalated with mudstone in the lower Luohe Formation; (c) Silty mudstone in the middle of the Luohe Formation; (d) Interbedding of fine sandstone and siltstone in the middle-upper Luohe Formation; (e) Sandstone intercalated with siltstone in the upper Luohe Formation; (f) Sandstone intercalated with mudstone in the middle of the Luohe Formation.

The Anding Formation is characterized by pale pink calcareous mudstone, which readily disintegrates upon contact with water. The Yijun Formation consists mainly of conglomerates, exceeding 50 m in thickness. These conglomerates are comprised of granite and metamorphic rocks, exhibiting diameters ranging from 3 centimeters to 8 centimeters and featuring calcareous cementation, along with a hard texture (Fig 20) [30]. As for the Luohe Formation, it predominantly comprises medium-fine, brownish-red sandstone, interspersed with similar-colored glutenite and conglomerate layers (Fig 21) [30]. The sandstone is primarily composed of quartz and feldspar, characterized by good sorting and loose cementation. The coarse-crystal conglomerate, featuring gravel diameters ranging from 0.2 m to 2 m, shares the same composition as the Yijun Formation’s conglomerate. Notably, the conglomerate exhibits substantial bedding thickness, varying from 35.83 m to 337.10 m, with an average of 163.20 m. The purplish-red hue of the conglomerate stems from the gravel often being encased in arenaceous-pelitic matter. The conglomerate layer displays poor sorting, loose cementation, and subrounded psephicity, with occasional instances of subangular and angular components.

In the weakly cemented Jurassic strata of the 1309 working face within the Guojiahe coal mine, the formation of rock beams is challenging, particularly given the expansive span and mining height of the area. Instead, these strata are more prone to experiencing shearing (cutting) failure. As the working face advances closer to the anticline axis, fracture lines that originate at the ends of the rock beams traverse through the Yan’an, Zhiluo, and Anding formations situated above the No. 3 coal seam. Unlike these formations, the Cretaceous Yijun Formation conglomerate is robust and substantial, preventing the fracture lines from penetrating through.

**Fig 22:**
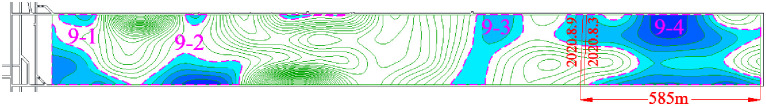
Low-resistance anomaly area in the water-bearing strata 90 m upon the No. 3 coal seam in the 1309 working face of the Guojiahe coal mine. The green line denotes the isogram of resistivity, while the blue area depicts the low-resistance area.

**Fig 23:**
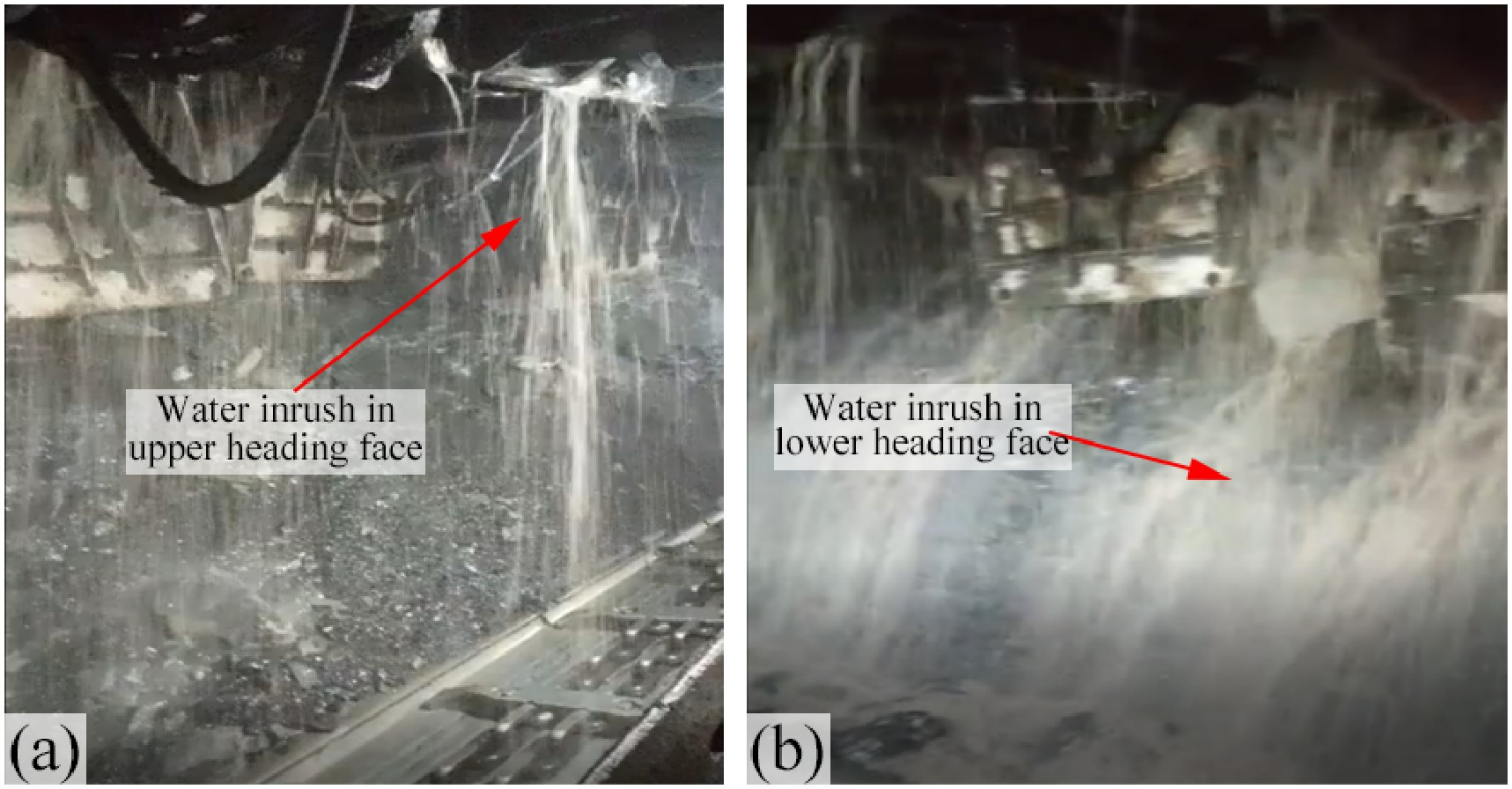
Water inrush from the head-on roof of the 1309 working face caused by fracture lines in the Guojiahe coal mine.

Due to significant mechanical disparities between the Yijun Formation conglomerate and the Anding Formation mudstone, these layers underwent separation along the stratum under the influence of mine pressure. This separation led to the complete collapse of the overlying strata within the region defined by the fracture lines and the separation surface. This collapse event resulted in the destruction of the fully mechanized mining supports (Fig 4a). Examination through transmission electron microscopy reveals the presence of a low-resistance anomaly near the anticline axis, signifying an increased abundance of water (Fig 22). The water-bearing Luohe Formation sandstone and Yijun Formation conglomerate were interconnected through structural fractures that formed along the anticline axis. Consequently, water within these strata surged into the head-on working face roof. This occurrence resembles a water curtain, signifying that the water inrush transpired across the entirety of the working face, with the water-inrush discharge escalating toward the lower gangway (Fig 23).

Following the collapse of the overlying strata, a broader fracture crack zone emerged, ushering in substantial quantities of rock fragments and gravels carried by the rushing water. The Anding Formation, constituted mainly of calcareous mudstone interlaced with some kaolinite, is prone to disintegration when exposed to water. This led to the inrushing water appearing turbid and pale in color.

#### Mechanism of roof water inrush from the head-on working face roof caused by structural fractures

On December 30, 2020, a significant water inrush event unfolded within the 1309 working face of the Guojiahe coal mine. This event resulted in the crushing of all fully mechanized mining supports, rendering them impossible to withdraw. Additionally, a collapse accident transpired in the lower gangway, prompting the need to reopen the open-off cut. The newly established open-off cut is situated 150 m away from the stop line, denoting the location of the 1309 working face on December 30, 2020.

Around 23:00 on August 20, 2021, water sprinkling was detected behind the protective frame within the 1309 working face, manifesting at a rate of approximately 10 m^3^ per hour. Subsequently, at approximately 7:00 on August 21, the inflow of water from the head-on working face roof surged to around 200 m^3^ per hour. In response, underground personnel were promptly evacuated to the surface. By approximately 7:20, the water inflow escalated even further, surpassing 1000 m^3^ per hour. Around 8:00, the air circulation within the 1309 working face was obstructed by the inundating water.

**Fig 24:**
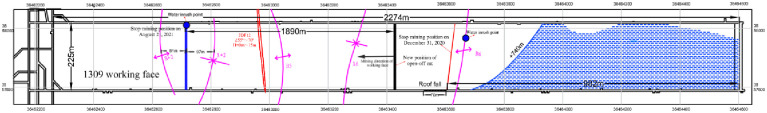
The water inrush point in the 1309 head-on working face in the Guojiahe coal mine, August 21, 2021.

The water inrush transpired when the cumulative mining length of the 1309 working face was approximately 1890 m. This position was approximately 97 m ahead of the syncline axis, in the direction of the working face’s advancement. Moreover, it was about 81 m distant from the anticline axis (as depicted in Fig 24). The water inrush incident persisted for about a day, with the peak water inflow rate reaching around 1500 m^3^ per hour. The cumulative water inflow throughout the event amounted to approximately 20000 m^3^.

Notably, the support resistance data indicated a regular mine pressure behavior both before and after the water inrush event. Unlike the prior water inrush incident, none of the fully mechanized mining supports were crushed, and there was no occurrence of a collapse accident. In this instance, no substantial rock fragments or gravels rushed into the head-on working face during the event. Additionally, the water’s appearance underwent a transformation from turbid to clear as the event progressed.

The fractures that had previously formed within the overlying strata were subsequently linked with structural fractures that developed within the anticline axis. This connection facilitated the creation of structural fracture passageways, which played a pivotal role in enabling the water inrush event. Within fold structures, the area around the anticline axis tends to exhibit a heightened development of tension fissures. These tension fissures serve a dual purpose, acting both as conduits for water movement and reservoirs for groundwater storage.

**Fig 25:**
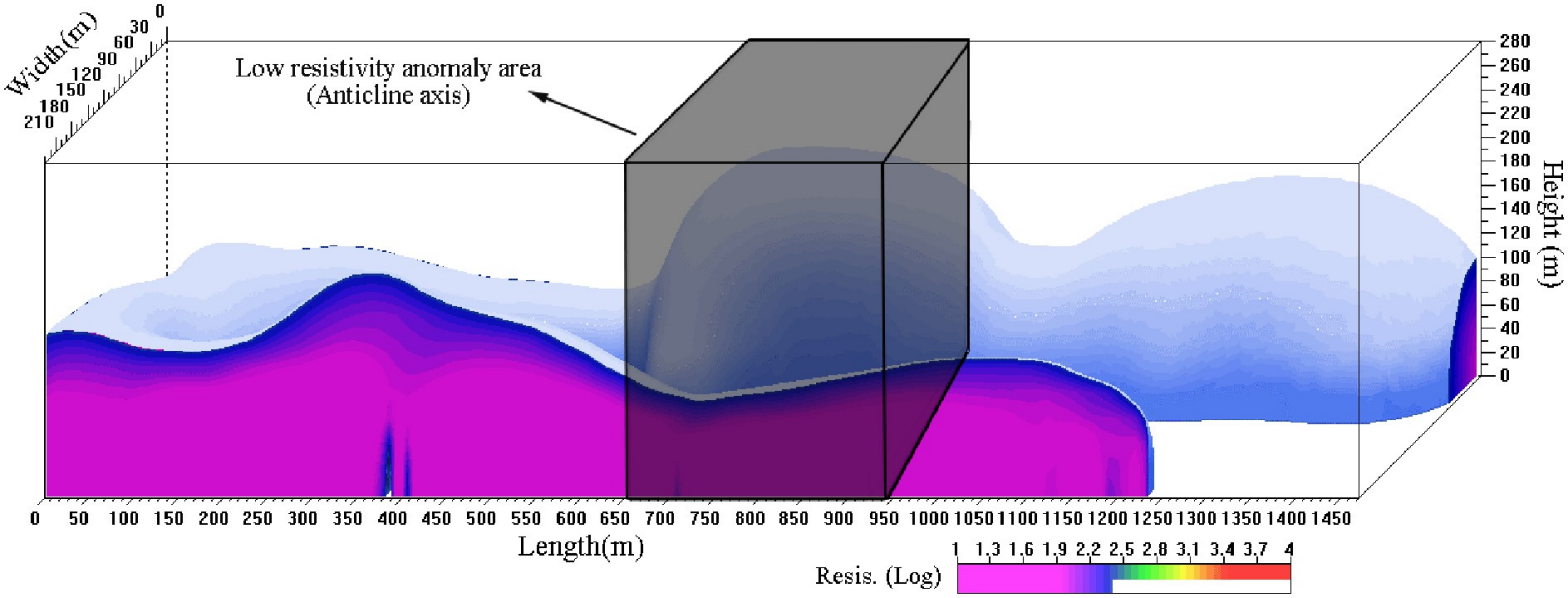
Low-resistance anomaly near the anticline axis.

The results of the 3D high-density electrical survey unveiled a conspicuous low-resistance anomaly in the vicinity of the anticline axis. This anomaly is indicative of a substantial abundance of water within this region (Fig 25).

**Fig 26:**
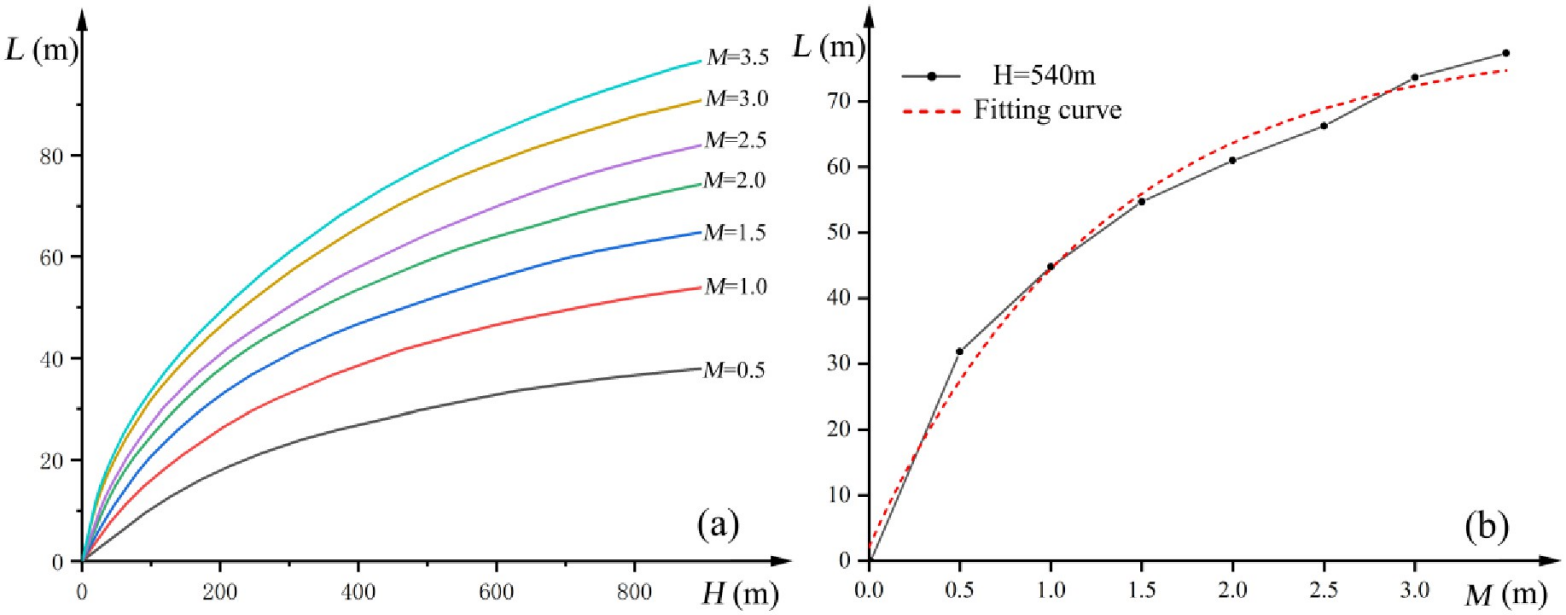
(a) The relationship between the advance movement distance of the overlying rock and the mining depth and thickness. (b) The relationship between the advance movement distance of overlying rock and mining thickness at a mining depth of 540 m.

As per the practical theory of mine pressure control, the process of coal seam mining triggers movements within the overlying strata, ultimately leading to their degradation until they are entirely disrupted. As the working face progresses, the overlying strata situated ahead of the head-on working face undergo destruction, and the extent of this destruction is closely tied to the thickness and depth of mining operations (Fig 26) [31].

In situations where the mining thickness exceeds 1.5 m, the distance over which the overlying strata are impacted by destruction increases by approximately 20 m for each additional meter in thickness. When the mining thickness reaches 3.5 m, the affected distance reaches approximately 100 m. For the 1309 working face in the Guojiahe coal mine, with a mining depth of 540 m. According to Fig 23a, a vertical line is drawn at a mining depth of 540 m to obtain a curve of the relationship between the advance movement of the overlying rock and mining thickness at a mining depth of *H* = 540 m (Fig 26b). The fitting formula (when the mining depth is 540 m) is as follows [32]:

$$L=\;23.447\text{ln}\left(m\right)+46.211$$ (7)

where *L* is the distance range affected by mine pressure, m and *m* is the mining thickness, m.

In the case of the 1309 working face in the Guojiahe coal mine, with a coal thickness of 10.3 m at a mining depth of 540 m, and considering a mining rate of 90% for a fully mechanized caving face (which yields a mining thickness of 9.3 m), the formula (*L* = 23.447*ln(*m*)+46.211) is used to calculate that approximately 98.5 m of the overlying strata ahead of the head-on working face are subjected to destruction. When the water inrush incident transpired, the head-on working face was positioned 81 m away from the anticline axis (in front of the head-on working face).

**Fig 27:**
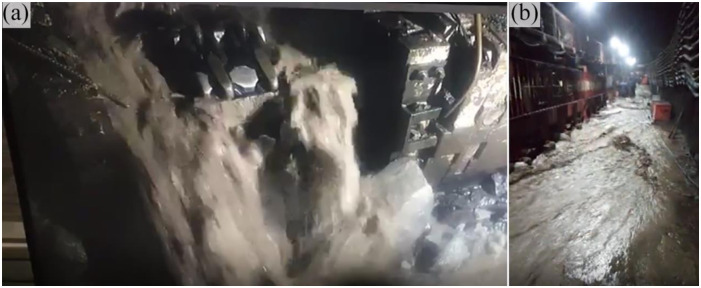
Water inrush from the roof of the 1309 head-on working face caused by structural fractures in the Guojiahe coal mine.

This signifies that the fractures developed within the disrupted overlying strata could be connected to the tensional structural fractures located near the anticline axis (within the 81 m distance in front of the head-on working face, which is smaller than the calculated 98.5 m destruction distance). Consequently, water within the structural fractures near the anticline axis could rapidly surge into the head-on working face roof, exhibiting a phenomenon akin to a transition from a point to a line. Initially, water inrush occurred at a single location on the head-on (referred to as punctate water inrush; as shown in Fig 27a), subsequently spreading across the entire head-on (forming a linear water inrush). With the passage of time, the water volume continuously increased, particularly towards the lower gangway (Fig 27b).

The fractures formed within the disrupted overlying strata are relatively narrow, and the water flow encountered difficulty in transporting massive rock fragments and gravels into the working face head-on. Initially, the water inrush led to the displacement of mud within the structural fractures, resulting in turbid water. As the phenomenon persisted, the water gradually cleared up.

#### Distinguishing method for the water-inrush passageways from the head-on working face roof

Based on the water-inrush mechanism from the head-on working face roof and the field data, water inflow pathways can be distinguished through the following aspects: variations in water inflow, duration of abnormal water discharge, peak water flow rate, water quality characteristics, and mine pressure behavior.

*Splitting zones type*. The influx of water experiences a gradual and prolonged rise before reaching its peak, resulting in extended periods of anomalous water surge. The upper limit of the influx is contingent upon the connectivity of the localized inrush zone with surface water bodies. Moreover, the escalation in water inflow from the frontal mining face substantially augments the water influx within the goaf. Notably, the water discharged during these episodes tends to be transparent, exhibiting minimal instances of sand or sediment expulsion. Evident manifestations of mine pressure are observable, characterized by frequent occurrences of roof and wall collapses within the tunnel. This heightened mine pressure exerts a conspicuous force on the support structures, occasionally leading to their deformation or even complete failure.

*Fracture line type*. The influx of water demonstrates a conspicuous escalation, achieving its peak rapidly and leading to abbreviated periods of atypical water surges. The upper threshold of this influx is contingent upon the abundance of water resources and the permeability of the aquiferous strata interconnected by fracture lines. With the amplification of water inflow from the frontal mining face, a slight augmentation in water influx is observed within the goaf. The discharged water during such occurrences typically appears turbid, accompanied by frequent ejections of sand and mud. Substantial indications of mine pressure behavior are evident, evidenced by recurrent incidents of roof and wall collapses within the tunnel. This pronounced mine pressure exerts a significant influence on the support structures, often resulting in their notable deformation or complete failure.

*Structural fracture type*. The water influx experiences a marked escalation, swiftly culminating in its maximum volume, accompanied by a brief span of irregular water surges. The zenith flow rate is governed by the aqueous capacity and pressure within the structural fractures. As the water inflow intensifies at the frontal mining face, there is no commensurate rise within the goaf. The water surges, typically characterized by turbidity, tend to transition to clarity, with infrequent occurrences of sand and mud eruptions. The mine pressure behavior remains within expected parameters, signifying the absence of roof or wall collapses. While the pressure exerted on the support structures is evident, widespread devastation of these supports is notably absent.

## Conclusions

After the overlying rock layer in the mining area is exposed, if it is in the form of bending (pulling) failure movement, the water passageway is mainly the water-conducting fracture zone, and the water inrush position of the roof is in the goaf. After the overlying rock layer in the mining area is exposed, if it is in the form of shearing (cutting) failure movement, the water passageways are mainly in the form of splitting zones type, fracture line type and structural fracture type, and the position of water inrush from the roof is head-on roof of working face. During the mining of Jurassic coal seams in the Ordos Basin, a movement pattern of shearing (cutting) failure movement is often formed, resulting in frequent water inrush from head-on roof of working face.

## Supporting information

S1 Table Tracer testing results. (DOCX)

S2 Table The shearing strengths of Jurassic sandstones. (DOCX)
