# Supplementary material for: Water-inrush mechanism from the head-on working face roof in a Jurassic coal seam in the Ordos Basin
Source: PLoS One. 2024 Mar 12;19(3):e0298399. doi: 10.1371/journal.pone.0298399 (PMC10931508; doi:10.1371/journal.pone.0298399)
Supplement: S1 Table — (DOCX) [file pone.0298399.s001.docx]

**S1 Table. Tracer testing results.**

| **Testing results for KI (detection limit: 0.002 mg/L)** | | | | | |
| --- | --- | --- | --- | --- | --- |
| **The first batch** | | **The second batch** | | **The third batch** | |
| **Sampling** | **Concentration**  **(mg/L)** | **Sampling** | **Concentration**  **(mg/L)** | **Sampling** | **Concentration**  **(mg/L)** |
| **No. 1** | 0 | **No. 1** | 0.027 | **No. 1** | 1.36 |
| **No. 2** | 0 | **No. 2** | 0.012 | **No. 2** | 1.36 |
| **No. 3** | 0 | **No. 3** | 0.004 | **No. 3** | 1.36 |
| **No. 4** | 0 | **No. 4** | 0.004 | **No. 4** | 0.453 |
| **No. 5** | 0 | **No. 5** | 0 | **No. 5** | 0.489 |
| **No. 6** | 0 | **No. 6** | 0 | **No. 6** | 1.22 |
| **No. 7** | 0 | **No. 7** | 0.006 | **No. 7** | 1.20 |
| **No. 8** | 0 | **No. 8** | 0.005 | **No. 8** | 0.505 |
| **No. 9** | 0 | **No. 9** | 0 | **No. 9** | 0.496 |
| **No. 10** | 0 | **No. 10** | 0.026 | **No. 10** | 2.09 |
| **No. 11** | 0 | **No. 11** | 0.029 | **No. 11** | 2.18 |
| **No. 12** | 0 | **No. 12** | 0.010 | **No. 12** | 0.562 |
| **No. 13** | 0 | **No. 13** | 0.009 | **No. 13** | 0.559 |
| **No. 14** | 0 | **No. 14** | 0.024 | **No. 14** | 0.569 |
| **No. 15** | 0 | **No. 15** | 0.026 | **No. 15** | 1.51 |
| **No. 16** | 0 | **No. 16** | 0.013 | **No. 16** | 1.50 |
| **No. 17** | 0 | **No. 17** | 0 |  |  |
| **No. 18** | 0 | **No. 18** | 0 |  |  |
|  |  | **No. 19** | 0.004 |  |  |
|  |  | **No. 20** | 0 |  |  |
