# Supplementary material for: Water-inrush mechanism from the head-on working face roof in a Jurassic coal seam in the Ordos Basin
Source: PLoS One. 2024 Mar 12;19(3):e0298399. doi: 10.1371/journal.pone.0298399 (PMC10931508; doi:10.1371/journal.pone.0298399)
Supplement: S2 Table — (DOCX) [file pone.0298399.s002.docx]

**S2 Table. The shearing strengths of Jurassic sandstones.**

| Lithology | Coarse sandstone | Medium sandstone | Fine sandstone |
| --- | --- | --- | --- |
| Shearing strength (Mpa) | 8.8 | 5.9 | 5.2 |
| Angle of internal friction (°) | 30.1 | 34.2 | 23.1 |
